# Incidence of noninvasive ventilation failure and mortality in patients with acute respiratory distress syndrome: a systematic review and proportion meta-analysis

**DOI:** 10.1186/s12890-024-02839-8

**Published:** 2024-01-22

**Authors:** Jie Wang, Jun Duan, Ling Zhou

**Affiliations:** 1 Department of Radiology, The First Affiliated Hospital of Chongqing Medical University, Youyi Road 1, Yuzhong District, 400016 Chongqing, China; 2 Department of Respiratory and Critical Care Medicine, The First Affiliated Hospital of Chongqing Medical University, Youyi Road 1, Yuzhong District, 400016 Chongqing, China; 3 Department of Medical Laboratory, Song Shan Hospital of Chongqing, 69 Renhe Xingguang Avenue, Yubei District, 401121 Chongqing, China

**Keywords:** Noninvasive ventilation; ARDS, Treatment failure, Mortality

## Abstract

**Background:**

Noninvasive ventilation (NIV) is commonly used in patients with acute respiratory distress syndrome (ARDS). However, the incidence and distribution of treatment failure are unclear.

**Methods:**

A comprehensive online search was conducted to select potentially eligible studies with reports of the rate of NIV failure in patients with ARDS. A manual search was also performed to identify additional studies. Data were extracted to calculate the pooled incidences of NIV failure and mortality. Based on oxygenation, the severity of the disease was classified as mild, moderate, or severe ARDS. Based on etiologies, ARDS was defined as being of pulmonary origin or extrapulmonary origin.

**Results:**

We enrolled 90 studies in this meta-analysis, involving 98 study arms. The pooled incidence of NIV failure was 48% (n = 5847, 95% confidence interval [CI]: 43–52%). The pooled incidence of ICU mortality was 29% (n = 2363, 95%CI: 22–36%), and that of hospital mortality was 33% (n = 2927, 95%CI: 27–40%). In patients with mild, moderate, and severe ARDS, the pooled incidence of NIV failure was 30% (n = 819, 95%CI: 21–39%), 51% (n = 1332, 95%CI: 43–60%), and 71% (n = 525, 95%CI: 62–79%), respectively. In patients with pulmonary ARDS, it was 45% (n = 2687, 95%CI: 39–51%). However, it was 30% (n = 802, 95%CI: 21–38%) in those with extrapulmonary ARDS. In patients with immunosuppression, the incidence of NIV failure was 62% (n = 806, 95%CI: 50–74%). However, it was 46% (n = 5041, 95%CI: 41–50%) in those without immunosuppression.

**Conclusions:**

Nearly half of patients with ARDS experience NIV failure. The incidence of NIV failure increases with increasing ARDS severity. Pulmonary ARDS seems to have a higher rate of NIV failure than extrapulmonary ARDS. ARDS patients with immunosuppression have the highest rate of NIV failure.

**Supplementary Information:**

The online version contains supplementary material available at 10.1186/s12890-024-02839-8.

## Introduction

Acute respiratory distress syndrome (ARDS) was first described by Ashbaugh et al. in 1967 [1]. Its features are acute onset of hypoxemia, bilateral opacities not fully explained by effusions, lobar/lung collapse or nodules, respiratory failure not fully explained by cardiac failure or fluid overload, and PaO_2_/FiO_2_ less than 300 mmHg [2]. The etiologies of ARDS include pneumonia, pancreatitis, abdominal infection, blood transfusion, and trauma [3, 4]. ARDS is classified as pulmonary or extrapulmonary according to its cause. Based on oxygenation, it can be classified as mild, moderate, or severe [5]. To relieve respiratory distress, respiratory support is commonly used in ARDS patients.

Physiological studies have shown that noninvasive ventilation (NIV) decreases the work of breathing and improves oxygenation in patients with ARDS [6]. In contrast with invasive mechanical ventilation, NIV preserves the ability to swallow, cough, and communicate verbally; avoids intubation-associated complications; and reduces the likelihood of nosocomial pneumonia. Therefore, NIV has been commonly used in patients with ARDS [7]. However, the incidence and distribution of NIV failure in ARDS population are unclear. Here, we report the incidence of NIV failure in ARDS patients and further clarify the distributions of NIV failure in different subgroups.

## Methods

This article reports the results of a systematic review and meta-analysis of NIV failure and mortality, focusing on patients with ARDS. It was performed in conformity with Preferred Reporting Items for Systematic Reviews and Meta-analysis statement [8].

### Search techniques and selection criteria

We searched PubMed, Web of Science, the Cochrane library, and some Chinese databases (CBM, Wanfang Data, and CNKI), without any language limitation, for pertinent research published before September 30, 2022. We also performed manual searches of the reference lists of the identified articles and pertinent reviews to identify additional relevant articles. The search used the following key words: (“noninvasive ventilation” OR “noninvasive mechanical ventilation” OR “noninvasive positive pressure ventilation” OR “NIV” OR “NPPV” OR “NIPPV” OR “continuous positive airway pressure” OR “CPAP” OR “noninvasive pressure support ventilation” OR “noninvasive oxygen” OR “noninvasive oxygenation” OR “mask ventilation” OR “nasal ventilation” OR “helmet ventilation”) and (“ARDS” OR “acute respiratory distress syndrome” OR “ALI” OR “acute lung injury” OR “acute respiratory failure” OR “acute hypoxemic respiratory failure” OR “hypoxemic respiratory failure”).

Studies were enrolled based on the following inclusion criteria: ARDS or ALI was diagnosed, adult patients were involved, and NIV was used as a first-line intervention. The following works were excluded: reviews, case reports, editorials, letters, and conference abstracts; articles with no available data for NIV failure; studies that used NIV as preoxygenation before intubation; and studies that used NIV as a ventilator weaning strategy.

### Data extraction and quality assessment

All studies were independently selected by two investigators (JW and JD). Any discrepancies were resolved by consensus. If the researchers failed to reach consensus, a third investigator (LZ) reviewed the data and made a determination. We extracted the data as follows: first author’s name, country, publication year, proportion of male patients, proportion of immunosuppressed patients, age, PaO_2_/FiO_2_, number of total patients, number of patients with ARDS, number of NIV failures in patients with ARDS, number of deaths in patients with ARDS, NIV mode, NIV interface, causes of ARDS, and the proportion of mild, moderate, and severe ARDS. NIV failure and mortality data were also collected.

We intended to report the incidence of treatment failure and mortality in ARDS patients who received NIV; thus, the quality of enrolled studies was assessed using Murad’s tool for non-comparative studies [9, 10]. This method includes five questions: (1) did the patients represent all of the cases seen by the medical center, (2) was the diagnosis correctly made, (3) were other important diagnosis excluded, (4) were all important data cited in the report, and (5) was the outcome correctly ascertained? Each question was assigned 1 (yes) or 0 (no) points. The quality of an enrolled study was classified as high, moderate, or low if the total scores were 5, 4, or ≤ 3, respectively.

### Statistical analysis

The data were analyzed using R (version 4.2.2). The Shapiro-Wilk test of normality was used to analyze the distribution of the incidence of NIV failure and mortality among the included studies. Values of p more than 0.05 were taken to indicate that the rate of the relevant event was normally distributed. If it was abnormally distributed, the data were transferred to normally distributed data using logarithmic transformation, sine transformation, or arc sine transformation. I^2^ was used to describe heterogeneity, where I^2^ ≥ 50% represents significant heterogeneity. A random-effects model was used to pool the data, and a subgroup analysis was performed to explore potential heterogeneity. If no heterogeneity was observed, a common-effects model (also known as a fixed-effects model) was used.

The mean-pooled incidences of treatment failure and mortality were estimated. The corresponding confidence intervals (95% CIs) were also estimated. Egger’s test was used to assess the possibility of publication bias [11]. A funnel plot (plot of treatment effect against trial precision) was created to visualize publication bias.

## Results

### Characteristics of the included studies

**Fig. 1:**
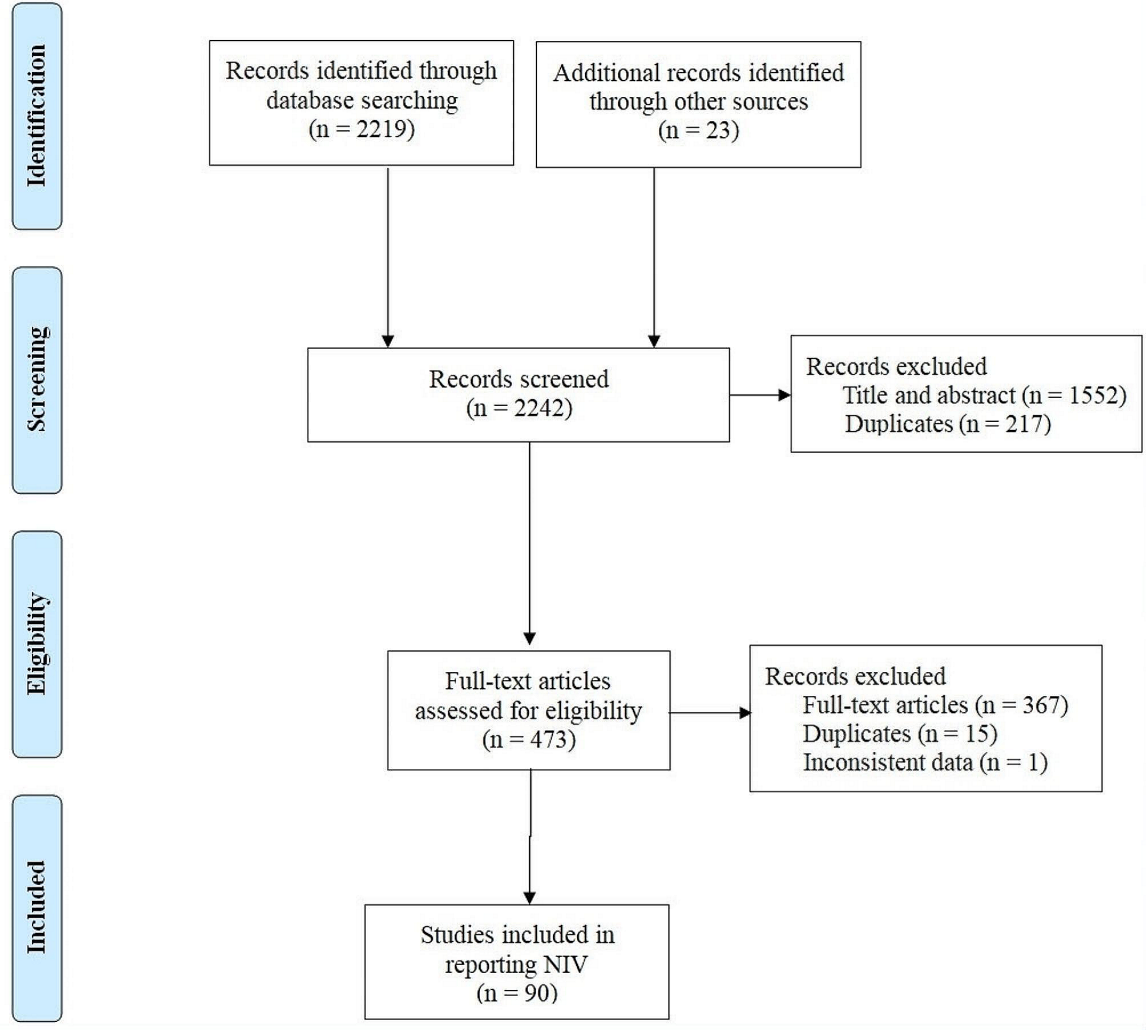
Flowchart of study selection

In all, 2219 studies were obtained using the search strategy, and 23 studies were identified from other sources (Fig. 1). After screening the titles and abstracts and reviewing full papers, we enrolled 90 studies involving 98 study arms. These studies were published between 1999 and 2022 (Table 1). Egger’s test (*p* = 0.69) and a funnel plot showed that there was no publication bias (Supplementary Fig. 1). The quality of most of the studies was high or moderate (Supplementary Fig. 2).

**Table 1:** Characteristics of the included studies

Author	Year	Country	Design	COVID-19	Male (%)	Immunosuppression (%)	Age	PaO_2_/FiO_2_	Definition of NIV failure	Patients with ARDS	NIV interface	NIV mode	NIV failure	ICU mortality	Hospital mortality
Rocker	1999	Canada	Observational	No	30%	NA	47 ± 23	102 ± 57	Intubation or death	10	Oronasal	BiPAP	5	3	3
Antonelli	2000	Italy	RCT	No	65%	100%	45 ± 19	129 ± 30	Intubation	8	Oronasal	BiPAP	3	3	NA
Hilbert	2000	France	Observational	No	55%	100%	45 ± 16	128 ± 32	Intubation	64	Oronasal	CPAP	48	44	NA
Delclaux	2000	France	RCT	No	61%	NA	56 (19–85)^#^	140 (59–228)^#^	Intubation	40	Oronasal	CPAP	15	9	12
Antonelli	2001	Italy	Observational	No	64%	10%	Success: 58 (16–96)^$^ Failure: 60 (13–86)^$^	Success: 119 ± 35 Failure: 120 ± 40	Intubation	86	Oronasal	BiPAP	44	26	NA
Confalonieri	2002	Italy	Observational	No	NA	100%	37 ± 9	122 ± 44	Intubation	24	Oronasal	BiPAP	8	6	NA
Ferrer	2003	Spain	RCT	No	55%	18%	61 ± 17	102 ± 21	Intubation	7	Oronasal	BiPAP	6	5	NA
Zhi	2003	China	Observational	No	42%	NA	40 ± 3	Success: 144 ± 15 Failure: 111 ± 8	Intubation or death	12	Oronasal or nasal	BiPAP	4	NA	4
Xu	2003	China	Observational	No	57%	NA	59 ± 12	Pul^*^: 147 ± 28 Extrapul^*^: 163 ± 46	Intubation	23	Oronasal	BiPAP	6	NA	NA
Rai	2004	India	Observational	No	64%	NA	68 (29–82)^$^	NA	Intubation	4	Oronasal	BiPAP	2	NA	NA
Li	2005	China	Observational	No	72%	NA	Pul^*^: 38 ± 12 Extrapul^*^: 34 ± 15	Pul^*^: 138 ± 29 Extrapul^*^: 141 ± 33	Intubation	18	Oronasal	BiPAP	8	NA	6
Gu	2005	China	Observational	No	69%	NA	43 (29–84)^$^	PaO_2_ 51 ± 5	Intubation	29	Oronasal or nasal	BiPAP	4	NA	4
Rana	2006	USA	Observational	No	61%	43%	Success: 60 (46–84) Failure: 64 (55–73)	Success: 147 (118–209) Failure: 112 (70–157)	Intubation	54	Oronasal	BiPAP or CPAP	38	NA	26
Antonelli	2007	Italy	Observational	No	63%	15%	Success: 53 (35–64) Failure: 60 (51–68)	Success: 116 ± 38 Failure: 105 ± 33	Intubation	147	Oronasal or helmet	BiPAP	68	41	53
Adda	2008	France	Observational	No	68%	100%	Success: 59 (47–66) Failure: 57 (47–67)	PaO_2_ success: 44 (38–51) PaO_2_ failure: 54 (41–59)	Intubation	32	Oronasal	BiPAP	31	NA	NA
Yoshida	2008	Japan	Observational	No	72%	NA	Success: 68 ± 12 Failure: 70 ± 10	Success: 122 ± 43 Failure: 125 ± 51	Intubation	47	Oronasal	BiPAP or CPAP	14	9	13
Domenighetti	2008	Switzerland	Observational	No	42%	NA	66 ± 8	104 ± 42	Intubation	12	Oronasal	BiPAP	4	5	NA
Agarwal	2009	India	Observational	No	43%	43%	42 ± 24	131 ± 46	Intubation	21	Oronasal	BiPAP	12	7	NA
Ding	2009	China	Observational	No	74%	42%	49 ± 17	123 ± 32	Intubation, death or withdrawal of therapy	31	Oronasal	BiPAP or CPAP	8	NA	NA
Zan	2009	China	Observational	No	68%	NA	58 (32–79)^$^	151 ± 19	Intubation	22	Oronasal	BiPAP	10	NA	9
Wang	2009	China	Observational	No	53%	NA	28 ± 2	229 ± 5	Intubation	21	Oronasal	BiPAP	6	NA	2
Gu	2010	China	Observational	No	65%	NA	38 (28–64)^$^	PaO_2_ 55 ± 4	Intubation or tracheotomy	40	Oronasal or nasal	BiPAP	9	0	0
Uçgun	2010	Turkey	Observational	No	90%	NA	45 ± 20	NA	Intubation	10	Oronasal	BiPAP	6	3	NA
Gristina	2011	Italy	Observational	No	59%	100%	60 ± 16	NA	NA	89	Oronasal or helmet	BiPAP	53	52	56
Bhadade	2011	India	Observational	No	74%	NA	NA	NA	Intubation	17	Oronasal	NA	11	NA	NA
Bai	2011	China	Observational	No	61%	12%	46 (34–60)	244 (211–290)	Intubation or death	24	NA	NA	11	9	NA
Kirakli	2011	Turkey	Observational	No	44%	NA	39 (24–52)	Survivor: 150 (98–232) Nonsurvivor: 67 (60–100)	Intubation	10	NA	NA	7	NA	6
Kikuchi	2011a	Japan	Observational	No	70%	NA	69 ± 17	PaO_2_ 79 ± 27	Intubation	17	Oronasal or nasal	BiPAP or CPAP	4	NA	NA
Kikuchi	2011b	Japan	Observational	No	51%	NA	58 ± 23	PaO_2_ 81 ± 43	Intubation	18	Oronasal or nasal	BiPAP or CPAP	6	NA	NA
Carrillo	2012a	Spain	Observational	No	59%	NA	62 ± 18	127 ± 34	Intubation or death	35	Oronasal or nasal	BiPAP	25	NA	13
Carrillo	2012b	Spain	Observational	No	77%	NA	72 ± 11	136 ± 37	Intubation or death	15	Oronasal or nasal	BiPAP	9	NA	7
Sharma	2012	India	Observational	No	52%	NA	48 ± 18	125 ± 78	Intubation	12	Oronasal	BiPAP	9	NA	NA
Zhan	2012	China	RCT	No	76%	24%	44 ± 14	225 ± 17	Intubation	21	Oronasal	BiPAP	1	1	1
Jin	2012	China	Observational	No	37%	NA	Yushu earthquake: 33 ± 20 Wenchuan earthquake: 52 ± 19	NA	Intubation	19	Oronasal	BiPAP	6	NA	NA
Zhi	2012	China	RCT	No	73%	NA	48 ± 17	139 ± 49	Intubation	15	Oronasal	BiPAP or CPAP	5	1	NA
Türkoğlu	2013	Turkey	Observational	No	69%	100%	45 (28–57)	104 (74–165)	Intubation	46	Oronasal	BiPAP	36	34	35
Wang	2013	China	Observational	No	54%	NA	Success: 70 (59–82) Failure: 60 (47–75)	Success: 192 ± 50 Failure: 170 ± 51	Intubation	24	Oronasal or nasal	NA	20	NA	NA
Thille	2013	France	Observational	No	66%	44%	Success: 58 ± 17 Failure: 63 ± 14	Success: 211 ± 86 Failure: 163 ± 92	Intubation	82	Oronasal	BiPAP	50	24	NA
Zhang	2014a	China	Observational	No	41%	NA	49 ± 10	127 ± 22	NIV intolerance or requirement of intubation or death	17	Oronasal	BiPAP	10	NA	NA
Zhang	2014b	China	Observational	No	53%	NA	48 ± 9	125 ± 24		17	Oronasal	BiPAP	7	NA	NA
Zhang	2014c	China	Observational	No	41%	NA	53 ± 9	132 ± 22		17	Oronasal	BiPAP	4	NA	NA
Verma	2014	India	Observational	No	52%	NA	NA	NA	Intubation	6	Oronasal	BiPAP	6	4	NA
Yu	2014	China	Observational	No	97%	NA	61 ± 7	126 ± 32	Intubation	64	Oronasal	NA	32	NA	2
Tsushima	2014	Japan	Observational	No	77%	NA	Survivor: 71 ± 14 Nonsurvivor: 81 ± 5	Survivor: 143 ± 62 Nonsurvivor: 122 ± 84	Intubation	47	Oronasal	BiPAP	17	NA	12
Sehgal	2015	India	Observational	No	34%	NA	27 (23–36)	180 (166–232)	Intubation	41	Oronasal	BiPAP	23	NA	19
Frat	2015	France	Observational	No	71%	36%	61 (49–68)	192 (158–251)	Intubation	23	Oronasal	BiPAP	8	NA	NA
Chawla	2016	India	Observational	No	62%	NA	49 ± 15	192 ± 51	Intubation	96	Oronasal	BiPAP	42	29	NA
Korkmaz	2016	Turkey	Observational	No	54%	71%	63 ± 16	145 ± 50	Intubation	28	Oronasal	BiPAP	17	NA	NA
Patel	2016a	USA	RCT	No	54%	36%	61 (56–71)	144 (90–223)	Intubation	39	Oronasal	BiPAP	24	NA	19
Patel	2016b	USA	RCT	No	55%	34%	58 (50–68)	118 (93–170)	Intubation	44	Helmet	BiPAP	8	NA	12
Meeder	2016	Netherlands	Observational	No	53%	11%	Success: 73 (28–87)^$^ Failure: 72 (41–88)^$^	Success: 296 (211–369) Failure: 207 (146–296)	Intubation	11	NA	NA	5	NA	NA
Ye	2016	China	Observational	No	60%	NA	54 ± 19	130 ± 46	Intubation	43	Oronasal	BiPAP	17	NA	22
Zhao	2016	China	Observational	No	60%	NA	47 ± 13	NA	Intubation	127	Oronasal or nasal	BiPAP	24	NA	9
Zeng	2016	China	Observational	No	57%	NA	Success: 58 ± 17 Failure: 65 ± 19	Success: 156 ± 12 Failure: 104 ± 10	Intubation	103	Oronasal	BiPAP	34	22	NA
Bellani	2017	Italy	Observational	No	60%	22%	Success: 67 (52–78) Failure: 63 (53–74)	Success: 171 ± 65 Failure: 145 ± 60	Intubation	349	NA	BiPAP or CPAP	131	79	94
Duan	2017a	China	Observational	No	70%	NA	Success: 65 ± 17 Failure: 66 ± 17	Success: 179 ± 83 Failure: 137 ± 65	Intubation	85	Oronasal	BiPAP or CPAP	61	NA	NA
Duan	2017b	China	Observational	No	62%	NA	Success: 65 ± 17 Failure: 67 ± 17	Success: 165 ± 63 Failure: 146 ± 68	Intubation	45	Oronasal	BiPAP or CPAP	27	NA	NA
Liu	2017	China	Observational	No	71%	100%	Success: 42 ± 10 Failure: 44 ± 11	Success: 114 ± 33 Failure: 111 ± 45	Intubation, death, or withdrawal of therapy	17	NA	BiPAP	8	NA	NA
Neuschwander	2017	France	Observational	No	64%	100%	57 (46–67)	NA	Intubation	387	Oronasal or nasal	NA	276	171	213
Liu	2017	Canada	Observational	No	60%	100%	56 ± 14	PaO_2_ 73 ± 38	Intubation or change to palliative care	41	Oronasal	BiPAP or CPAP	28	NA	NA
Kumar	2018	India	Observational	No	49%	NA	40 ± 3	197 ± 74	Intubation	35	Oronasal	NA	15	7	NA
Hong	2018	China	Observational	No	74%	NA	77 ± 7	141 ± 60	Intubation	64	Oronasal	BiPAP	46	NA	33
Briones-Claudett	2018	Ecuador	Observational	No	65%	NA	68 ± 22	PaO_2_ 77 ± 11	Intubation	38	Oronasal	BiPAP	13	11	NA
Wang	2018	China	Observational	No	68%	NA	68 ± 12	Success: 198 ± 74 Failure: 168 ± 63	Intubation	56	Oronasal	BiPAP or CPAP	20	NA	14
He	2019	China	RCT	No	67%	9%	53 ± 18	232 ± 35	Intubation	102	Oronasal	BiPAP	9	7	7
Bajaj	2019	India	Observational	No	46%	NA	53 ± 17	143 ± 94	Intubation	27	Oronasal	BiPAP	11	7	NA
Paternoster	2020	Italy	Observational	No	36%	NA	62 ± 10	108 ± 21	Intubation	11	Helmet	CPAP	3	NA	2
Satou	2020	Japan	Observational	No	75%	NA	68 ± 19	Success: 137 ± 69 Failure: 126 ± 67	Intubation or death	68	Oronasal	BiPAP or CPAP	16	NA	13
Liengswangwong	2020	Thailand	Observational	No	49%	NA	Success: 75 ± 13 Failure: 73 ± 14	PaO_2_ success: 122 ± 38 PaO_2_ Failure: 125 ± 60	Intubation	5	NA	NA	5	NA	NA
Ding	2020	China	Observational	No	65%	NA	Success: 47 ± 9 Failure: 54 ± 11	Success: 125 ± 41 Failure: 119 ± 19	Intubation	20	Oronasal	BiPAP or CPAP	9	1	1
Menzella	2020	Italy	Observational	Yes	71%	NA	67 ± 11	120 ± 42	Intubation	79	Oronasal	BiPAP	41	NA	30
Carrillo	2020	Spain	Observational	No	52%	NA	67 ± 17	141 ± 36	Intubation or death	262	Oronasal	BiPAP	184	NA	NA
Pagano	2020	Italy	Observational	Yes	72%	NA	Success: 70 ± 11 Failure: 68 ± 14	Success: 143 ± 91 Failure: 167 ± 72	Intubation	18	NA	CPAP	8	NA	11
Shen	2020	China	Observational	No	44%	100%	Success: 39 (27–55) Failure: 41 (30–54)	Success: 146 (114–204) Failure: 153 (103–228)	Intubation	70	Oronasal	BiPAP	44	39	NA
Duca	2020a	Italy	Observational	Yes	86%	NA	70 (62–79)	131 (97–190)	Intubation or death	71	Helmet	CPAP	65	NA	54
Duca	2020b	Italy	Observational	Yes	71%	NA	75 (59–80)	87 (53–120)	Intubation or death	7	Helmet	BiPAP	4	NA	4
Tonelli	2020	Italy	Observational	No	67%	NA	71 (66–81)	125 (101–170)	Intubation or death	15	Oronasal	BiPAP	7	NA	NA
Liu	2020	China	Observational	No	53%	40%	58 ± 12	65 ± 19	Intubation	15	Oronasal	BiPAP	10	NA	NA
Wang	2020	China	Observational	Yes	69%	NA	64 ± 7	268 ± 6	Intubation	32	NA	BiPAP	6	NA	8
Simioli	2021	Italy	Observational	Yes	86%	NA	64 ± 23	95 ± 57	Intubation	29	Oronasal or helmet	BiPAP or CPAP	3	3	NA
Drescher	2021	USA	Observational	No	51%	13%	Success: 65 (49–71) Failure: 67 (57–75)	Success: 198 (149–298) Failure: 187 (106–235)	Intubation	18	Oronasal	BiPAP	15	NA	NA
Koga	2021	Japan	Observational	No	68%	NA	78 (69–84)	141 (97–186)	Intubation, death or crossover to HFNC	10	Oronasal	BiPAP or CPAP	7	NA	NA
Carpagnano	2021	Italy	Observational	Yes	73%	NA	Survivor: 64 ± 14 Nonsurvivor: 75 ± 13	Survivor: 226 ± 76 Nonsurvivor: 137 ± 54	Death	61	NA	BiPAP or CPAP	25	25	NA
Menzella	2021	Italy	Observational	Yes	71%	NA	67 ± 11	120 ± 42	Intubation or death	79	Oronasal	BiPAP	41	20	NA
Briones	2021	Ecuador	Observational	No	66%	NA	71 ± 19	192 ± 40	Intubation	5	Oronasal	BiPAP	2	NA	1
Zhao	2021	China	Observational	Yes	75%	NA	68 (62–76)	NA	Meet the criteria of intubation	24	Oronasal	NA	16	NA	14
Zhu	2021	China	Observational	No	62%	NA	Success: 57 ± 13 Failure: 63 ± 15	Success: 145 ± 23 Failure: 122 ± 20	Intubation or death	180	NA	NA	27	NA	NA
Ramirez	2022	Italy	Observational	Yes	80%	5%	61 (53–71)	98 (74–137)	Intubation or death	90	Oronasal	CPAP	35	NA	17
Lazzeri	2022	Italy	Observational	Yes	75%	NA	Success: 60 ± 13 Failure: 70 ± 13	Success: 123 (110–145) Failure: 82 (66–103)	Intubation	75	Oronasal	BiPAP	31	NA	23
Sun	2022	China	Observational	No	64%	NA	Success: 66 (59–76) Failure: 65 (57–77)	Success: 236 ± 53 Failure: 194 ± 78	Meet the criteria of intubation	131	Oronasal	BiPAP or CPAP	64	NA	NA
Chiumello	2022	Italy	Observational	Yes	77%	NA	58 (52–64)	264 (204–301)	NA	104	Oronasal or helmet	BiPAP or CPAP	20	NA	13
Duan	2022a	China	Observational	No	69%	8%	Success: 63 ± 16 Failure: 64 ± 16	Success: 178 ± 74 Failure: 153 ± 60	Need for intubation	311	Oronasal or nasal	BiPAP or CPAP	153	NA	NA
Duan	2022b	China	Observational	No	67%	19%	Success: 61 ± 17 Failure: 61 ± 16	Success: 147 ± 104 Failure: 137 ± 85	Need for intubation	143	Oronasal or nasal	BiPAP or CPAP	86	NA	NA
Jurjević	2022	Croatia	Observational	Yes	57%	NA	66 ± 11	137 ± 57	Intubation	58	Oronasal	BiPAP or CPAP	15	NA	NA
Chacko	2022	India	Observational	Yes	84%	NA	53 ± 12	161 ± 80	Intubation or death	209	Oronasal	BiPAP	91	NA	NA
Isaac	2022	India	Observational	Yes	83%	NA	54 ± 12	NA	Intubation or death	168	Oronasal	BiPAP or CPAP	39	28	NA
Tetaj	2022	Italy	Observational	Yes	65%	NA	60 (48–73)	NA	Intubation or death	224	Oronasal or helmet	BiPAP or CPAP	64	NA	43
Yaroshetskiy	2022	Russia	Observational	Yes	56%	NA	72 (62–80)	Success: 131 (92–230) Failure: 90 (70–120)	Meet the criteria of intubation	80	Oronasal	BiPAP	57	NA	57

NA = not available, NIV = noninvasive ventilation, BiPAP = bi-level positive airway pressure, CPAP = continuous positive airway pressure, RCT = randomized control trial

^#^Data are reported as medians and 5th to 95th percentiles

^$^Data are reported as medians and ranges

*Pul; pulmonary; Extrapul: extrapulmonary

### NIV failure and mortality in ARDS patients

**Fig. 2:**
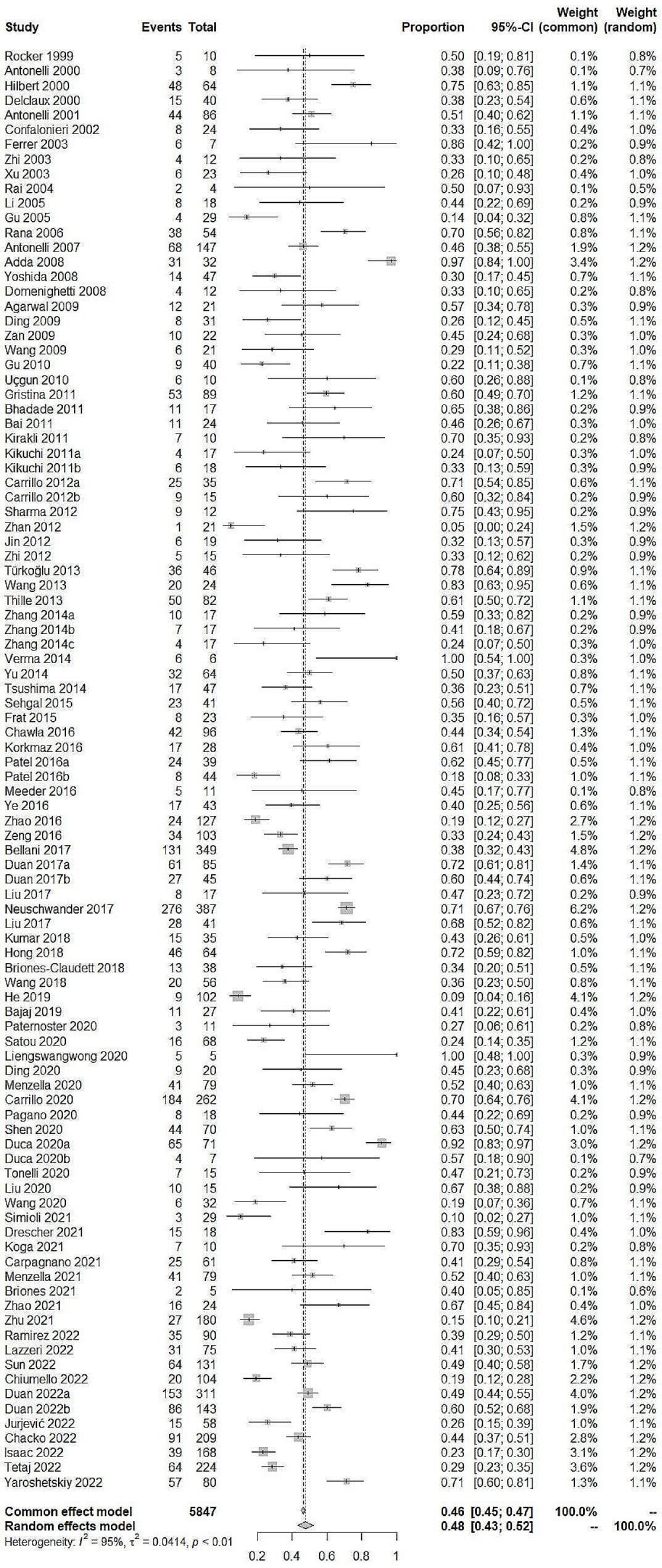
Pooled incidence of NIV failure

**Fig. 3:**
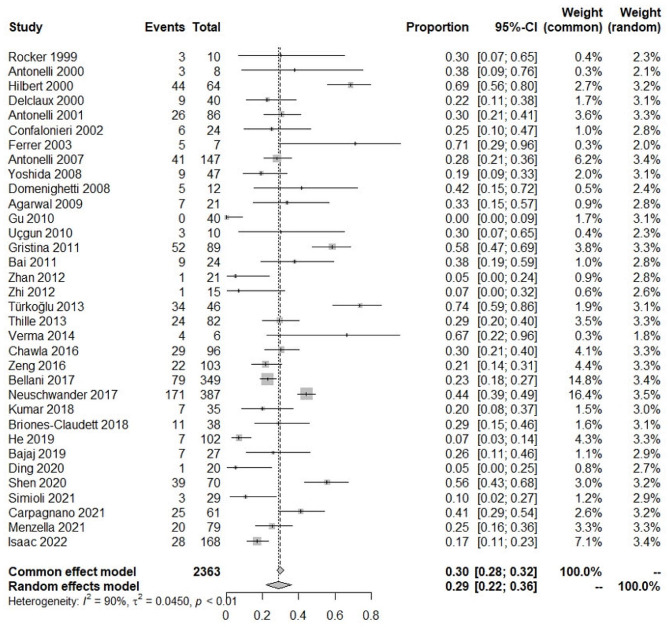
Pooled incidence of ICU mortality

**Fig. 4:**
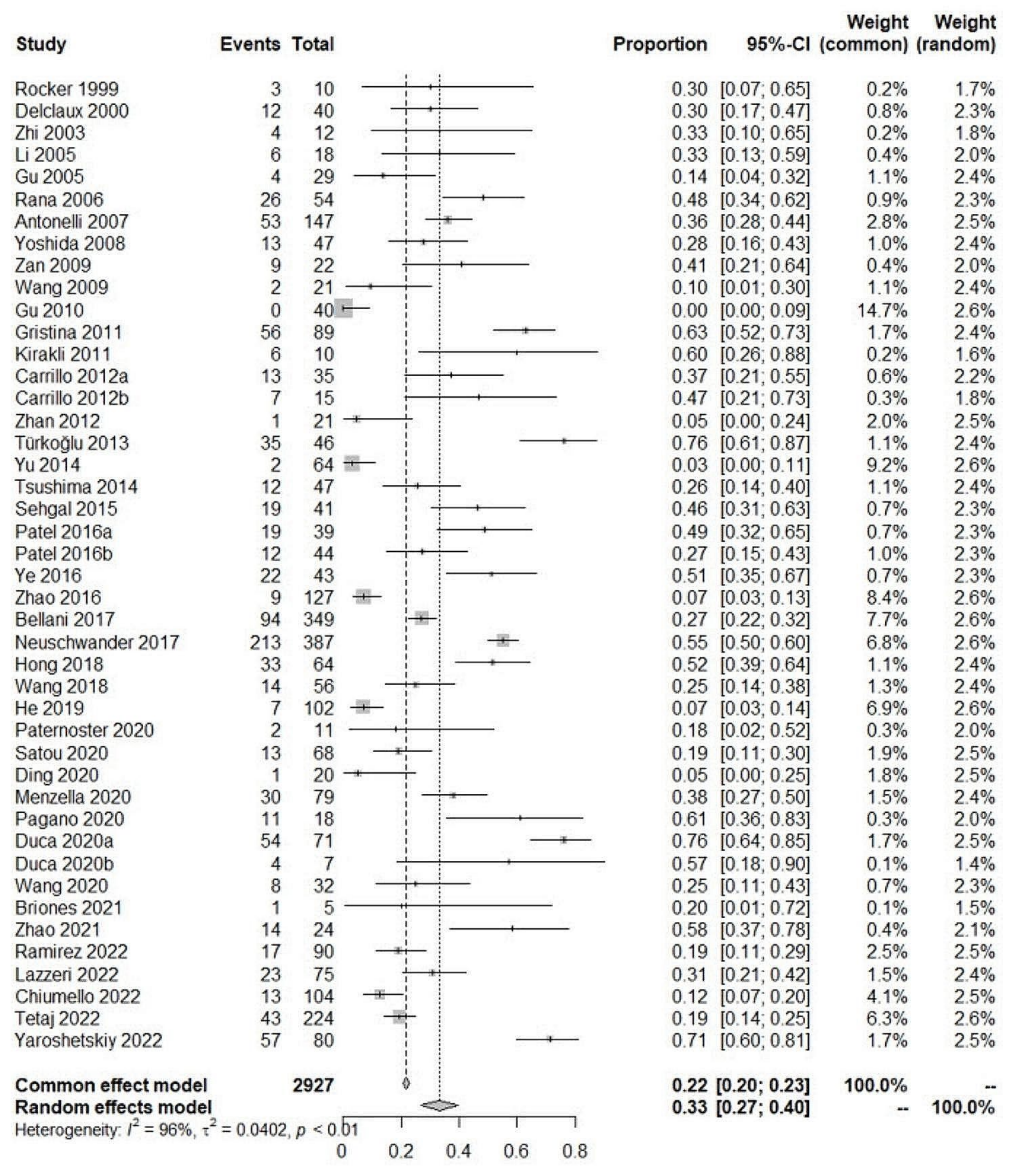
Pooled incidence of hospital mortality

In total, 5847 ARDS patients were enrolled in studies reporting NIV failure (Fig. 2). Significant heterogeneity was found between studies (I^2^ = 95%). Therefore, a random-effects model was selected. The pooled incidence of NIV failure was 48% (95%CI: 43–52%). In all, 2363 ARDS patients were enrolled in studies reporting ICU mortality (Fig. 3). The pooled incidence of ICU mortality was 29% (95%CI: 22–36%). In total, 2927 ARDS patients were enrolled in studies reporting hospital mortality (Fig. 4). The pooled incidence of hospital mortality was 33% (95%CI: 27–40%).

### NIV failure in different subgroups

**Fig. 5:**
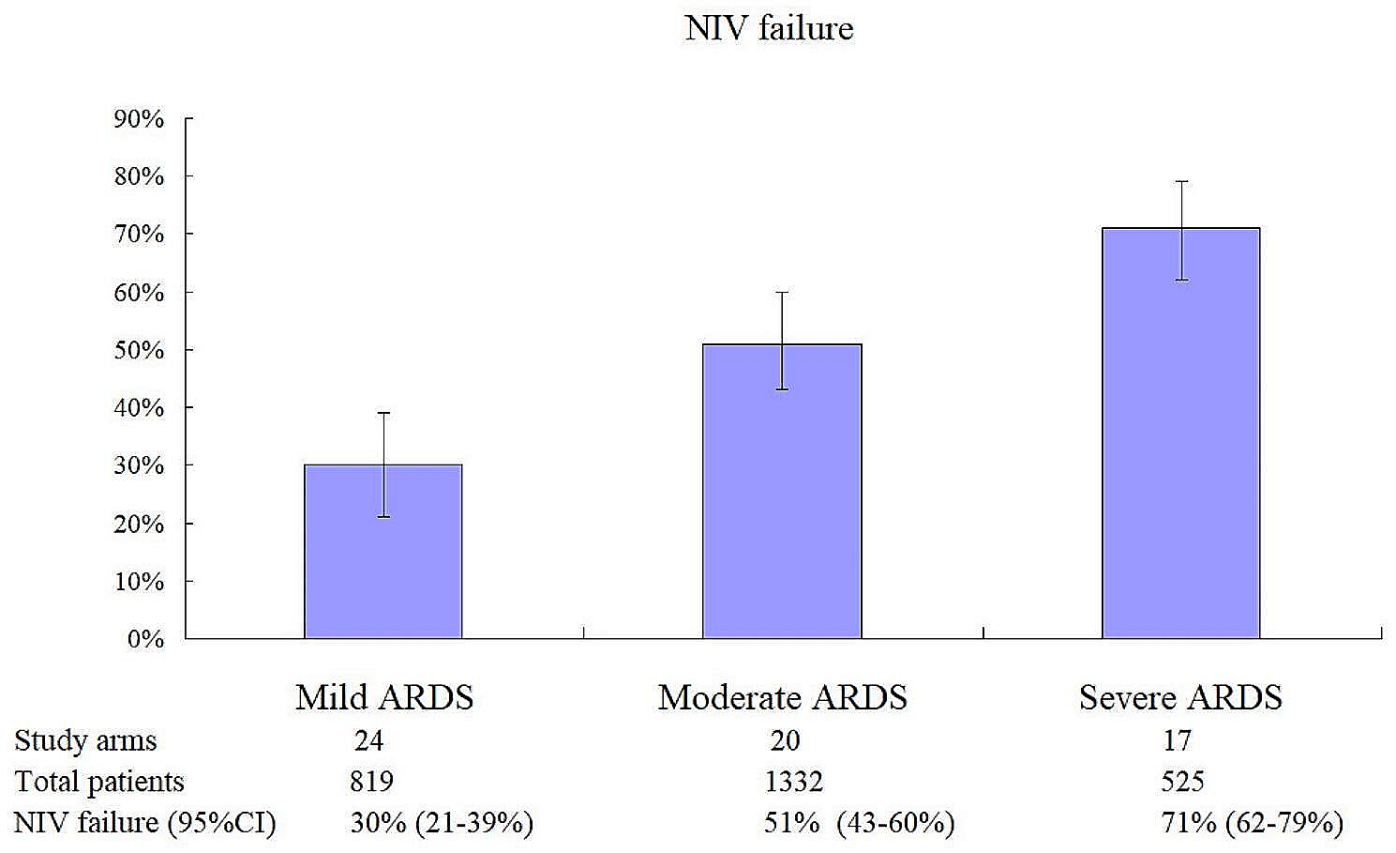
Pooled incidence of NIV failure in patients with mild, moderate, and severe ARDS

In all, 24, 20, and 17 study arms reported the rate of NIV failure in patients with mild, moderate, and severe ARDS, respectively (Fig. 5). The pooled incidence of NIV failure was 30% (95%CI: 21–39%), 51% (95%CI: 43–60%), and 71% (95%CI: 62–79%) in these ARDS groups, respectively.

**Fig. 6:**
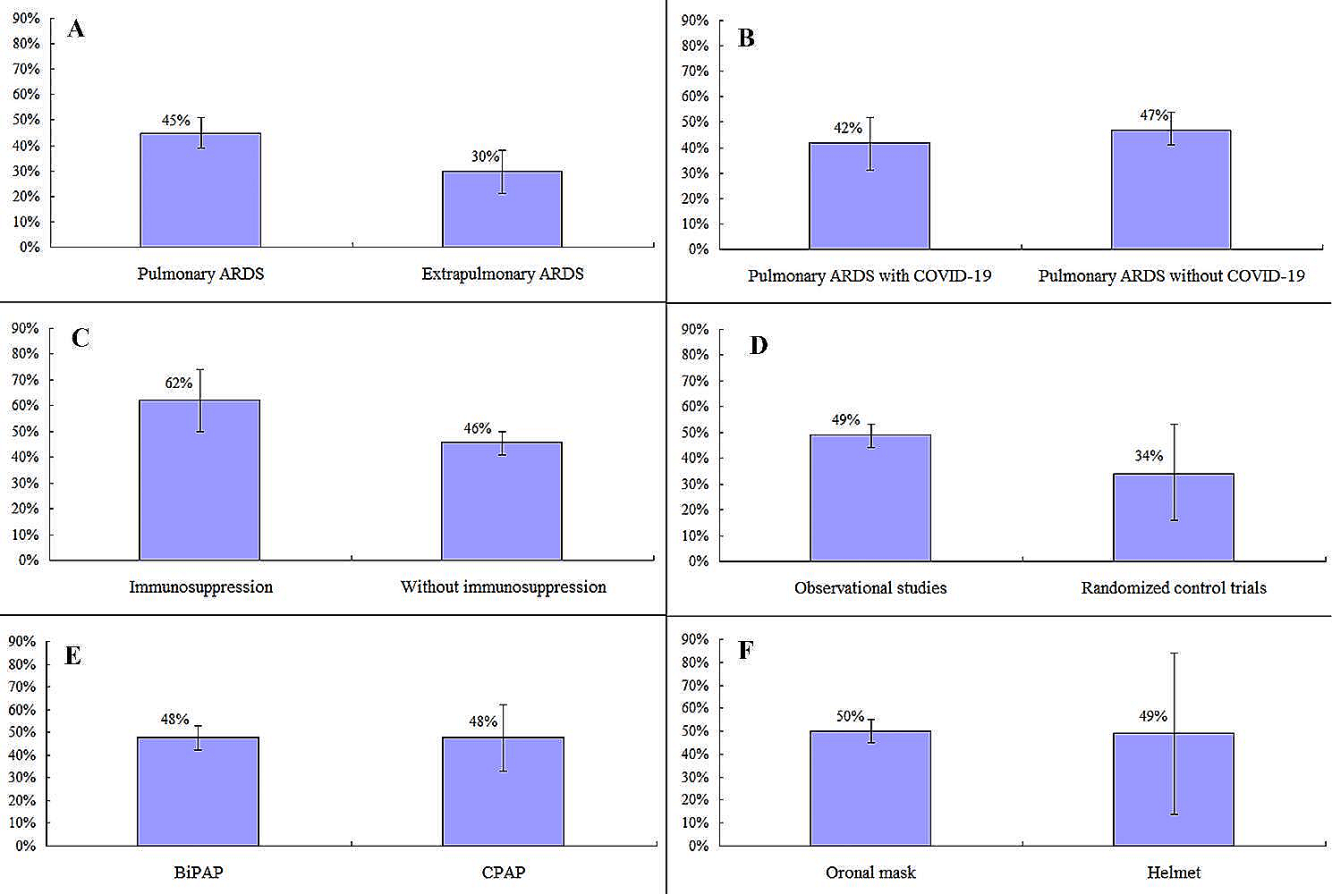
Pooled incidence of NIV failure in different subgroups

There were 46 study arms that involved 2687 patients with pulmonary ARDS that reported NIV failure (Supplementary Fig. 3), and 19 study arms that involved 802 patients with extrapulmonary ARDS that reported NIV failure (Supplementary Fig. 4). The pooled incidence of NIV failure was 45% (95%CI: 39–51%) and 30% (21–38%) in patients with pulmonary and extrapulmonary ARDS, respectively (Fig. 6A). For pulmonary ARDS in particular, the pooled incidence of NIV failure was 42% (95%CI: 31–52%) and 47% (95%CI: 41–54%) in patients with and without COVID-19, respectively (Fig. 6B, supplementary Figs. 5 and 6).

The pooled incidence of NIV failure was 62% (95%CI: 50–74%) and 46% (95%CI: 41–50%) in patients with and without immunosuppression, respectively (Fig. 6C, supplementary Figs. 7 and 8). NIV failure was 49% (95%CI: 44–53%) in observational studies and 34% (95%CI: 16–53%) in randomized control trials (Fig. 6D, supplementary Figs. 9 and 10). In patients who were ventilated using BiPAP, the NIV failure was 48%, the same as in those ventilated via CPAP (Fig. 6E, supplementary Figs. 11 and 12).

**Fig. 7:**
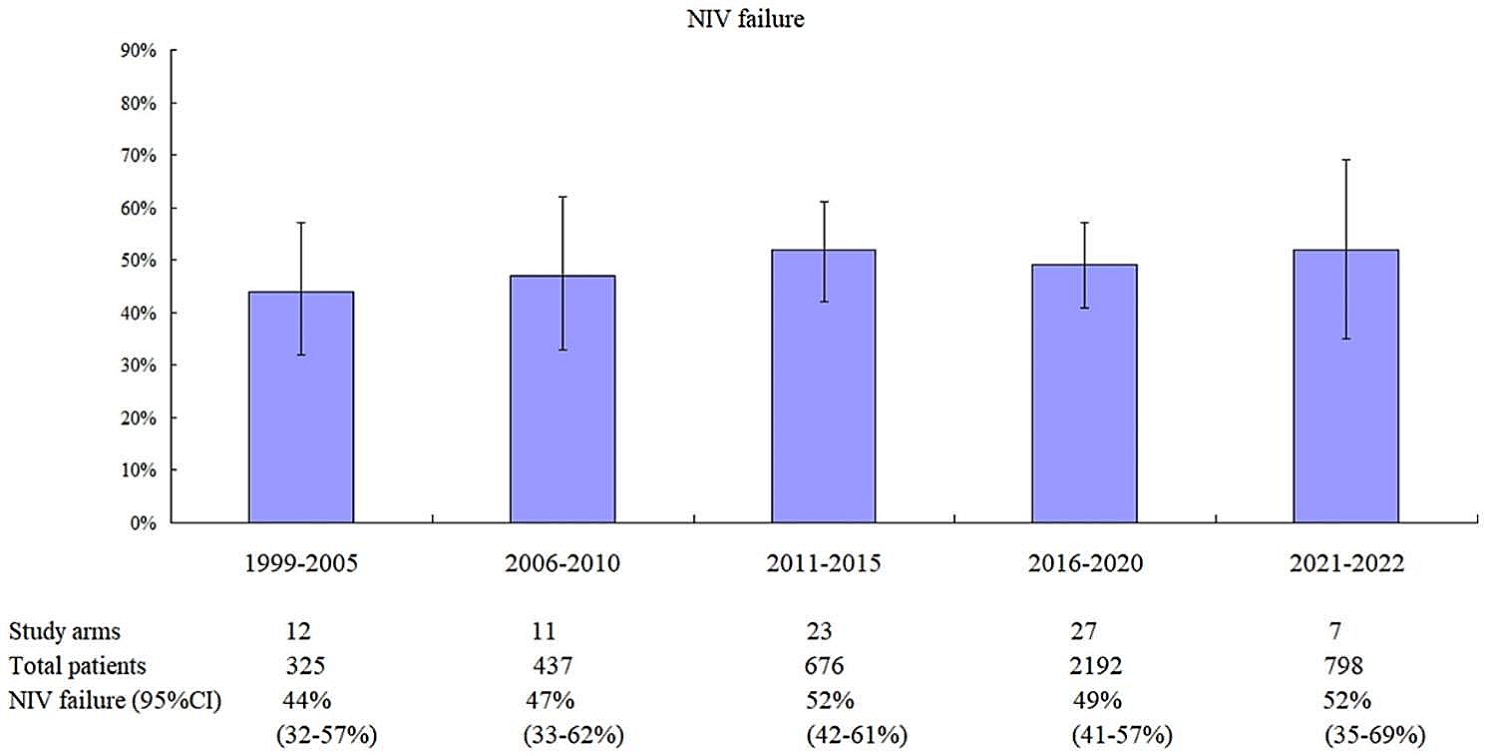
Pooled incidence of NIV failure in different timeframes

The pooled incidence of NIV failure was 44% (95%CI: 32–57%) before 2005 (Fig. 7). Between 2006 and 2010, it was 47% (33–62%). However, this did not change after 2010 when patients with COVID-19 were excluded (52% for 2011–2015, 49% for 2016–2020, and 52% for 2021 and 2022).

## Discussion

This meta-analysis shows that the incidence of NIV failure was high in patients with ARDS. A gradual increase in NIV failure was found in ARDS patients with an increase in disease severity. Patients with pulmonary ARDS had a higher rate of NIV failure than those with extrapulmonary ARDS. ARDS patients with immunosuppression had the highest failure rate.

An international, multicenter, prospective cohort study reported that 15.5% of patients with ARDS had received NIV [6]. This indicates that the use of NIV in patients with ARDS is not rare. However, ARDS is a risk factor for NIV failure in patients who have acute respiratory failure [12–14]. In our analyses, nearly half of the ARDS patients experienced NIV failure. ARDS patients commonly developed excessive inspiratory effort and high transpulmonary pressures; this can lead the patient to self-inflicted lung injury (P-SILI) [15, 16]. Therefore, P-SILI greatly contributes to the high rate of NIV failure in ARDS patients. Esophageal pressure monitoring can detect the risk for P-SILI, which can help identify NIV failure early [17].

This meta-analysis shows that pulmonary ARDS leads to a higher rate of NIV failure than extrapulmonary ARDS, in line with a previous study [18]. Pulmonary ARDS is mainly caused by pneumonia, and extrapulmonary ARDS is mainly caused by sepsis [19, 20]. In patients with pulmonary ARDS, consolidation in chest CT scans is greater, and the response to the lung recruitment maneuver is worse than that in patients with extrapulmonary ARDS [21, 22]. This may be the reason for the lower NIV failure rate in patients with extrapulmonary ARDS. However, our study was a meta-analysis and the demographic information between two groups could not be comparable. Studies are required to further clarify this issue.

It is challenging to avoid intubation in immunocompromised patients with acute respiratory failure [23]. Patients with immunosuppression were more likely to receive NIV as a first-line therapy [24]. Relative to conventional oxygen therapy, use of NIV reduces the rate of intubation in patients with immunosuppression [25]. However, in our analyses, the pooled incidence of NIV failure in the immunocompromised group was 62%, the highest of all subgroups. Patients who experienced NIV failure had a higher likelihood of death in hospital than those who directly received intubation [26]. Therefore, the early identification of high-risk patients followed by the early application of intubation would be an alternative solution to reduce mortality.

This study had several limitations. First, there were various definitions of NIV failure among the studies considered. Some studies defined it as intubation, whereas others defined it as intubation, death, or crossover to a high-flow nasal cannula. However, because it was commonly defined as intubation, the incidence of NIV failure may have been overestimated. Second, only four study arms, involving 133 patients, could be used to pool the incidence of helmet NIV failure. As helmet NIV shows much more favorable outcomes than oronasal NIV, the pooled incidence of helmet NIV failure may be overestimated [27]. Third, the incidence of NIV failure was high in the ARDS population. This does not imply that patients with ARDS cannot obtain benefits from NIV. In the future, randomized controlled trials should be performed to further investigate this issue.

## Conclusions

Nearly half of ARDS patients experience NIV failure. With increasing ARDS severity, the pooled incidence of NIV failure increased. Patients with pulmonary ARDS seem to experience more NIV failure than those with extrapulmonary ARDS. ARDS patients with immunosuppression may be at highest risk for NIV failure.

### Electronic supplementary material

Below is the link to the electronic supplementary material.

**Supplementary Material 1**: **Supplementary material 1**. Enrolled studies for meta-analysis. **Supplementary Fig. 1**. Funnel plot of the proportion of NIV failure. **Supplementary Fig. 2**. Quality assessment of enrolled studies. **Supplementary Fig. 3**. Pooled incidence of NIV failure in patients with pulmonary ARDS. **Supplementary Fig. 4**. Pooled incidence of NIV failure in patients with extra-pulmonary ARDS. **Supplementary Fig. 5**. Pooled incidence of NIV failure in pulmonary ARDS patients with COVID-19. **Supplementary Fig. 6**. Pooled incidence of NIV failure in pulmonary ARDS patients without COVID-19. **Supplementary Fig. 7**. Pooled incidence of NIV failure in patients with immunosuppression. **Supplementary Fig. 8**. Pooled incidence of NIV failure in patients without immunosuppression. **Supplementary Fig. 9**. Pooled incidence of NIV failure in observational studies. **Supplementary Fig. 10**. Pooled incidence of NIV failure in randomized control trials. **Supplementary Fig. 11**. Pooled incidence of NIV failure in patients who used BiPAP. **Supplementary Fig. 12**. Pooled incidence of NIV failure in patients who used CPAP. **Supplementary Fig. 13**. Pooled incidence of NIV failure in patients who used an oronasal mask. **Supplementary Fig. 14**. Pooled incidence of NIV failure in patients who used a helmet.

## Data Availability

The datasets analyzed during current study are available from the corresponding author on reasonable request.

## Abbreviations

NA: Not available
NIV: Noninvasive ventilation
BiPAP: Bi-level positive airway pressure
CPAP: Continuous positive airway pressure
ARDS: Acute respiratory distress syndrome
CI: Confidence interval
P-SILI: Patient self-inflicted lung injury
