# Supplementary material for: Incidence of noninvasive ventilation failure and mortality in patients with acute respiratory distress syndrome: a systematic review and proportion meta-analysis
Source: BMC Pulm Med. 2024 Jan 22;24:48. doi: 10.1186/s12890-024-02839-8 (PMC10802073; doi:10.1186/s12890-024-02839-8)
Supplement: Supplementary file 1 — Supplementary Material 1: Supplementary material 1. Enrolled studies for meta-analysis. Supplementary Fig. 1. Funnel plot of the proportion of NIV failure. Supplementary Fig. 2. Quality assessment of enrolled studies. Supplementary Fig. 3. Pooled incidence of NIV failure in patients with pulmonary ARDS. Supplementary Fig. 4. Pooled incidence of NIV failure in patients with extra-pulmonary ARDS. Supplementary Fig. 5. Pooled incidence of NIV failure in pulmonary ARDS patients with COVID-19. Supplementary Fig. 6. Pooled incidence of NIV failure in pulmonary ARDS patients without COVID-19. Supplementary Fig. 7. Pooled incidence of NIV failure in patients with immunosuppression. Supplementary Fig. 8. Pooled incidence of NIV failure in patients without immunosuppression. Supplementary Fig. 9. Pooled incidence of NIV failure in observational studies. Supplementary Fig. 10. Pooled incidence of NIV failure in randomized control trials. Supplementary Fig. 11. Pooled incidence of NIV failure in patients who used BiPAP. Supplementary Fig. 12. Pooled incidence of NIV failure in patients who used CPAP. Supplementary Fig. 13. Pooled incidence of NIV failure in patients who used an oronasal mask. Supplementary Fig. 14. Pooled incidence of NIV failure in patients who used a helmet. [file 12890_2024_2839_MOESM1_ESM.doc]

**Supplementary materials**

Supplementary material 1. Enrolled studies for meta-analysis.

Supplementary Figure 1. Funnel plot of the proportion of NIV failure.

Supplementary Figure 2. Quality assessment of enrolled studies.

Supplementary Figure 3. Pooled incidence of NIV failure in patients with pulmonary ARDS.

Supplementary Figure 4. Pooled incidence of NIV failure in patients with extra-pulmonary ARDS.

Supplementary Figure 5. Pooled incidence of NIV failure in pulmonary ARDS patients with COVID-19.

Supplementary Figure 6. Pooled incidence of NIV failure in pulmonary ARDS patients without COVID-19.

Supplementary Figure 7. Pooled incidence of NIV failure in patients with immunosuppression.

Supplementary Figure 8. Pooled incidence of NIV failure in patients without immunosuppression.

Supplementary Figure 9. Pooled incidence of NIV failure in observational studies.

Supplementary Figure 10. Pooled incidence of NIV failure in randomized control trials.

Supplementary Figure 11. Pooled incidence of NIV failure in patients who used BiPAP.

Supplementary Figure 12. Pooled incidence of NIV failure in patients who used CPAP.

Supplementary Figure 13. Pooled incidence of NIV failure in patients who used an oronasal mask.

Supplementary Figure 14. Pooled incidence of NIV failure in patients who used a helmet.

**Supplementary material 1. Enrolled studies for meta-analysis**

1. Rocker GM, Mackenzie MG, Williams B, et al. Noninvasive positive pressure ventilation: successful outcome in patients with acute lung injury/ARDS. Chest 1999; 115:173-177
2. Antonelli M, Conti G, Bufi M, et al. Noninvasive ventilation for treatment of acute respiratory failure in patients undergoing solid organ transplantation: a randomized trial. JAMA 2000; 283:235-241
3. Hilbert G, Gruson D, Vargas F, et al. Noninvasive continuous positive airway pressure in neutropenic patients with acute respiratory failure requiring intensive care unit admission. Crit Care Med 2000; 28:3185-3190
4. Delclaux C, L'Her E, Alberti C, et al. Treatment of acute hypoxemic nonhypercapnic respiratory insufficiency with continuous positive airway pressure delivered by a face mask: A randomized controlled trial. JAMA 2000; 284:2352-2360
5. Antonelli M, Conti G, Moro ML, et al. Predictors of failure of noninvasive positive pressure ventilation in patients with acute hypoxemic respiratory failure: a multi-center study. Intensive Care Med 2001; 27:1718-1728
6. Confalonieri M, Calderini E, Terraciano S, et al. Noninvasive ventilation for treating acute respiratory failure in AIDS patients with Pneumocystis carinii pneumonia. Intensive Care Med 2002; 28:1233-1238
7. Ferrer M, Esquinas A, Leon M, et al. Noninvasive ventilation in severe hypoxemic respiratory failure: a randomized clinical trial. Am J Respir Crit Care Med 2003; 168:1438-1444
8. Zhi RC, Xie MY, Zhou JT. Noninvasive positive pressure ventilation in early acute respiratory distress syndrome, Chin J Respir Crit Care Med, 2003, 2(2): 97-99
9. Xu SC, Huang YF, Wang XY, et al. Effect of noninvasive positive pressure ventilation on treatment of acute respiratory distress syndrome, Chinese Critical Care Medicine, 2003, 16(6):354-357
10. Rai SP, Panda BN, Upadhyay KK. Noninvasive Positive Pressure Ventilation in Patients with Acute Respiratory Failure. Med J Armed Forces India 2004; 60:224-226
11. Li J, Wang JZ, Ding M, et al. Noninvasive positive pressure ventilation in the patients with acute lung injury/acute respiratory distress syndrome, Chin J Crit Care Med, 2005, 25(1): 25-27.
12. Gu MP. Nursing of NIPPV therapy for severe acute pancreatitis with ARDS, Journal of Chongqing Medical University, 2005, 30(3): 477-479
13. Rana S, Jenad H, Gay PC, et al. Failure of non-invasive ventilation in patients with acute lung injury: observational cohort study. Crit Care 2006; 10:R79
14. Antonelli M, Conti G, Esquinas A, et al. A multiple-center survey on the use in clinical practice of noninvasive ventilation as a first-line intervention for acute respiratory distress syndrome. Crit Care Med 2007; 35:18-25
15. Adda M, Coquet I, Darmon M, et al. Predictors of noninvasive ventilation failure in patients with hematologic malignancy and acute respiratory failure. Crit Care Med 2008; 36:2766-2772
16. Yoshida Y, Takeda S, Akada S, et al. Factors predicting successful noninvasive ventilation in acute lung injury. J Anesth 2008; 22:201-206
17. Domenighetti G, Moccia A, Gayer R. Observational case-control study of non-invasive ventilation in patients with ARDS. Monaldi Arch Chest Dis 2008; 69:5-10
18. Agarwal R, Handa A, Aggarwal AN, et al. Outcomes of noninvasive ventilation in acute hypoxemic respiratory failure in a respiratory intensive care unit in north India. Respir Care 2009; 54:1679-1687
19. Ding L, Zhan QY, Luo ZJ, et al. [Non-invasive positive pressure ventilation in patients with acute respiratory distress syndrome: a prospective cohort study]. Zhongguo Wei Zhong Bing Ji Jiu Yi Xue 2009; 21:613-616
20. Zan HF, Xu Z, Yang X. Noninvasive positive pressure ventilation in the patients with acute respiratory distress syndrome, Sichuan Medical Journal, 2009, 30(2): 201-203
21. Wang Y, Yue SC. Noninvasive ventilatory support in the management of acute lung injury after thoracic trauma by complex critical ventilator, Journal of Dalian Medical University, 2009, 31(4): 303-307
22. Gu MP, Ding F, Fan J. Clinical evaluation of noninvasive positive pressure ventilation in multiple injury patients with acute lung injury and acute respiratory distress syndrome, Journal of Chongqing Medical University, 2010, 35(12):1884-1887
23. Uçgun I, Yildirim H, Metintaş M, et al. The efficacy of non-invasive positive pressure ventilation in ARDS: a controlled cohort study. Tuberk Toraks 2010; 58:16-24
24. Gristina GR, Antonelli M, Conti G, et al. Noninvasive versus invasive ventilation for acute respiratory failure in patients with hematologic malignancies: a 5-year multicenter observational survey. Crit Care Med 2011; 39:2232-2239
25. Bhadade RR, de Souza RA, Harde MJ, et al. Clinical characteristics and outcomes of patients with acute lung injury and ARDS. J Postgrad Med 2011; 57:286-290
26. Bai L, Gu L, Cao B, et al. Clinical features of pneumonia caused by 2009 influenza A(H1N1) virus in Beijing, China. Chest 2011; 139:1156-1164
27. Kirakli C, Tatar D, Cimen P, et al. Survival from severe pandemic H1N1 in urban and rural Turkey: a case series. Respir Care 2011; 56:790-795
28. Kikuchi T, Toba S, Sekiguchi Y, et al. Protocol-based noninvasive positive pressure ventilation for acute respiratory failure. J Anesth 2011; 25:42-49
29. Carrillo A, Gonzalez-Diaz G, Ferrer M, et al. Non-invasive ventilation in community-acquired pneumonia and severe acute respiratory failure. Intensive Care Med 2012; 38:458-466
30. Sharma S, Agarwal R, Aggarwal AN, et al. A survey of noninvasive ventilation practices in a respiratory ICU of North India. Respir Care 2012; 57:1145-1153
31. Zhan Q, Sun B, Liang L, et al. Early use of noninvasive positive pressure ventilation for acute lung injury: a multicenter randomized controlled trial. Crit Care Med 2012; 40:455-460
32. Jin XD, Zhou YF, Wang B, et al. Clinical significance of critically injured acute respiratory distress syndrome patients early treated with non-invasive ventilation management strategy in Yushu earthquake, Modern Preventive Medicine, 2012, 39(1): 226-229
33. Zhi RC, Li YH, Huang CP, et al. Noninvasive positive pressure ventilation in acute lung injury and acute respiratory distress syndrome: A randomized Controlled Study, Chin J Respir Crit Care Med, 2012, 11(6): 522-527
34. Türkoğlu M, Erdem GU, Suyanı E, et al. Acute respiratory distress syndrome in patients with hematological malignancies. Hematology 2013; 18:123-130
35. Wang S, Singh B, Tian L, et al. Epidemiology of noninvasive mechanical ventilation in acute respiratory failure--a retrospective population-based study. BMC Emerg Med 2013; 13:6
36. Thille AW, Contou D, Fragnoli C, et al. Non-invasive ventilation for acute hypoxemic respiratory failure: intubation rate and risk factors. Crit Care 2013; 17:R269
37. Zhang J, Cao J, Feng J, et al. A study of noninvasive positive-pressure mechanical ventilation in the treatment of acute lung injury with a complex critical care ventilator. J Int Med Res 2014; 42:788-798
38. Verma AK, Mishra M, Kant S, et al. Noninvasive mechanical ventilation: An 18-month experience of two tertiary care hospitals in north India. Lung India 2013; 30:307-311
39. Yu KY, Zhao L, Chen Z, et al. Noninvasive positive pressure ventilation for the treatment of acute respiratory distress syndrome following esophagectomy for esophageal cancer: a clinical comparative study. J Thorac Dis 2013; 5:777-782
40. Tsushima K, Yokoyama T, Matsumura T, et al. The potential efficacy of noninvasive ventilation with administration of a neutrophil elastase inhibitor for acute respiratory distress syndrome. J Crit Care 2014; 29:420-425
41. Sehgal IS, Chaudhuri S, Dhooria S, et al. A study on the role of noninvasive ventilation in mild-to-moderate acute respiratory distress syndrome. Indian J Crit Care Med 2015; 19:593-599
42. Frat JP, Brugiere B, Ragot S, et al. Sequential application of oxygen therapy via high-flow nasal cannula and noninvasive ventilation in acute respiratory failure: an observational pilot study. Respir Care 2015; 60:170-178
43. Chawla R, Mansuriya J, Modi N, et al. Acute respiratory distress syndrome: Predictors of noninvasive ventilation failure and intensive care unit mortality in clinical practice. J Crit Care 2016; 31:26-30
44. Korkmaz Ekren P, Basarik Aydogan B, Gurgun A, et al. Can fiberoptic bronchoscopy be applied to critically ill patients treated with noninvasive ventilation for acute respiratory distress syndrome? Prospective observational study. BMC Pulm Med 2016; 16:89
45. Patel BK, Wolfe KS, Pohlman AS, et al. Effect of Noninvasive Ventilation Delivered by Helmet vs Face Mask on the Rate of Endotracheal Intubation in Patients With Acute Respiratory Distress Syndrome: A Randomized Clinical Trial. JAMA 2016; 315:2435-2441
46. Meeder AM, Tjan DH, van Zanten AR. Noninvasive and invasive positive pressure ventilation for acute respiratory failure in critically ill patients: a comparative cohort study. J Thorac Dis 2016; 8:813-825
47. Ye L, Wang J, Xu X, et al. Noninvasive ventilation on mortality of acute respiratory distress syndrome. J Phys Ther Sci 2016; 28:2284-2288
48. Zhao X, Huang W, Li J, et al. Noninvasive Positive-Pressure Ventilation in Acute Respiratory Distress Syndrome in Patients With Acute Pancreatitis: A Retrospective Cohort Study. Pancreas 2016; 45:58-63
49. Zeng WX, Jiang WQ, Wen MY, et al. Analysis of clinical effect of noninvasive positive pressure ventilation in the treatment of acute respiratory distress syndrome, Chin Crit Care Med, 2016, 28(6): 539-542
50. Bellani G, Laffey JG, Pham T, et al. Noninvasive Ventilation of Patients with Acute Respiratory Distress Syndrome. Insights from the LUNG SAFE Study. Am J Respir Crit Care Med 2017; 195:67-77
51. Duan J, Han X, Bai L, et al. Assessment of heart rate, acidosis, consciousness, oxygenation, and respiratory rate to predict noninvasive ventilation failure in hypoxemic patients. Intensive Care Med 2017; 43:192-199
52. Liu X, Xie Z, Teng H, et al. [Efficacy of noninvasive ventilation on treatment of ARDS caused by severe pneumonia after kidney transplantation]. Zhonghua Wei Zhong Bing Ji Jiu Yi Xue 2017; 29:994-998
53. Neuschwander A, Lemiale V, Darmon M, et al. Noninvasive ventilation during acute respiratory distress syndrome in patients with cancer: Trends in use and outcome. J Crit Care 2017; 38:295-299
54. Liu J, Bell C, Campbell V, et al. Noninvasive Ventilation in Patients With Hematologic Malignancy. J Intensive Care Med 2017:885066617690725
55. Kumar SS, Selvarajan Chettiar KP, Nambiar R. Etiology and Outcomes of ARDS in a Resource Limited Urban Tropical Setting. J Natl Med Assoc 2018; 110:352-357
56. Hong Y, Duan J, Bai L, et al. Noninvasive ventilation failure in pneumonia patients >/=65years old: The role of cough strength. J Crit Care 2018; 44:149-153
57. Briones-Claudett KH, Esquinas Rodriguez A, Briones-Claudett MH, et al. Use of noninvasive mechanical ventilation with pressure support guaranteed with average volume in de novo hypoxaemic respiratory failure. A pilot study. Anaesthesiol Intensive Ther 2018; 50:283-290
58. Wang H, Dong SM, Zhang SC, et al. Factors for the failure of noninvasive positive pressure ventilation for patients with mild or moderate acute respiratory distress syndrome induced by sepsis, Clinical Focus, 2018, 33(11): 966-973
59. He H, Sun B, Liang L, et al. A multicenter RCT of noninvasive ventilation in pneumonia-induced early mild acute respiratory distress syndrome. Crit Care 2019; 23:300
60. Bajaj A, Kumar S, Inamdar AH, et al. Noninvasive ventilation in acute hypoxic respiratory failure in medical intensive care unit: A study in rural medical college. Int J Crit Illn Inj Sci 2019; 9:36-42
61. Paternoster G, Sartini C, Pennacchio E, et al. Awake pronation with helmet continuous positive airway pressure for COVID-19 acute respiratory distress syndrome patients outside the ICU: A case series. Med Intensiva (Engl Ed) 2020; 46:65-71
62. Satou T, Imamura H, Mochiduki K, et al. Efficacy of protocol-based non-invasive positive pressure ventilation for acute respiratory distress syndrome: a retrospective observational study. Acute Med Surg 2020; 7:e465
63. Liengswangwong W, Yuksen C, Thepkong T, et al. Early detection of non-invasive ventilation failure among acute respiratory failure patients in the emergency department. BMC Emerg Med 2020; 20:80
64. Ding L, Wang L, Ma W, et al. Efficacy and safety of early prone positioning combined with HFNC or NIV in moderate to severe ARDS: a multi-center prospective cohort study. Crit Care 2020; 24:28
65. Menzella F, Fontana M, Salvarani C, et al. Efficacy of tocilizumab in patients with COVID-19 ARDS undergoing noninvasive ventilation. Crit Care 2020; 24:589
66. Carrillo A, Lopez A, Carrillo L, et al. Validity of a clinical scale in predicting the failure of non-invasive ventilation in hypoxemic patients. J Crit Care 2020; 60:152-158
67. Pagano A, Porta G, Bosso G, et al. Non-invasive CPAP in mild and moderate ARDS secondary to SARS-CoV-2. Respir Physiol Neurobiol 2020; 280:103489
68. Shen J, Hu Y, Zhao H, et al. Risk factors of non-invasive ventilation failure in hematopoietic stem-cell transplantation patients with acute respiratory distress syndrome. Ther Adv Respir Dis 2020; 14:1753466620914220
69. Duca A, Memaj I, Zanardi F, et al. Severity of respiratory failure and outcome of patients needing a ventilatory support in the Emergency Department during Italian novel coronavirus SARS-CoV2 outbreak: Preliminary data on the role of Helmet CPAP and Non-Invasive Positive Pressure Ventilation. EClinicalMedicine 2020; 24:100419
70. Tonelli R, Fantini R, Tabbì L, et al. Early Inspiratory Effort Assessment by Esophageal Manometry Predicts Noninvasive Ventilation Outcome in De Novo Respiratory Failure. A Pilot Study. Am J Respir Crit Care Med 2020; 202:558-567
71. Liu S, Zhu HD, Yu XZ, et al. Efficacy of high-flow nasal cannula oxygen and noninvasive positive pressure ventilation in the initial treatment of pulmonary moderate and severe acute respiratory distress syndrome. Journal of Clinical Emergency (China), 2020, 21(3): 181-187
72. Wang CP, Huang XL, Luo XB, et al. The comparative study of high-flow nasal cannula oxygen therapy and non-invasive ventilation in treatment of acute respiratory distress syndrome induced by severe coronavirus disease 2019, Chinese Journal of Respiratory and Critical Care Medicine, 2020, 21 (1): 20-24.
73. Simioli F, Annunziata A, Langella G, et al. Early Prone Positioning and Non-Invasive Ventilation in a Critical COVID-19 Subset. A Single Centre Experience in Southern Italy. Turk Thorac J 2021; 22:57-61
74. Drescher GS, Al-Ahmad MM. Analysis of Noninvasive Ventilation in Subjects With Sepsis and Acute Respiratory Failure. Respir Care 2021; 66:1063-1073
75. Koga Y, Kaneda K, Fujii N, et al. Association between increased nonaerated lung weight and treatment failure in patients with de novo acute respiratory failure: Difference between high-flow nasal oxygen therapy and noninvasive ventilation in a multicentre retrospective study. J Crit Care 2021; 65:221-225
76. Carpagnano GE, Buonamico E, Migliore G, et al. Bilevel and continuous positive airway pressure and factors linked to all-cause mortality in COVID-19 patients in an intermediate respiratory intensive care unit in Italy. Expert Rev Respir Med 2021; 15:853-857
77. Menzella F, Barbieri C, Fontana M, et al. Effectiveness of noninvasive ventilation in COVID-19 related-acute respiratory distress syndrome. Clin Respir J 2021; 15:779-787
78. Briones Claudett KH, Esquinas Rodriguez A, Briones Claudett MH, et al. Non-invasive mechanical ventilation with average volume-assured pressure support. Results according to the aetiology of acute respiratory failure. Anaesthesiol Intensive Ther 2021; 53:403-410
79. Zhao Z, Cao H, Cheng Q, et al. [Effect of noninvasive positive pressure ventilation and high-flow nasal cannula oxygen therapy on the clinical efficacy of coronavirus disease 2019 patients with acute respiratory distress syndrome]. Zhonghua Wei Zhong Bing Ji Jiu Yi Xue 2021; 33:708-713
80. Zhu LL, Li P. Analysis of related factors affecting the efficacy of noninvasive positive pressure ventilation in the treatment of sepsis-induced ARDS, Journal of Hebei Medical University, 2021, 42(3): 281-285
81. Ramirez GA, Bozzolo EP, Castelli E, et al. Continuous positive airway pressure and pronation outside the Intensive Care Unit in COVID-19 acute respiratory distress syndrome. Minerva Med 2022; 113:281-290
82. Lazzeri C, Bonizzoli M, Batacchi S, et al. Coupling of right ventricular function to pulmonary circulation as an independent predictor for non invasive ventilation failure in SARSCoV 2-related acute respiratory distress syndrome. Am Heart J Plus 2022; 18:100178
83. Sun W, Luo Z, Cao Z, et al. A combination of the APACHE II score, neutrophil/lymphocyte ratio, and expired tidal volume could predict non-invasive ventilation failure in pneumonia-induced mild to moderate acute respiratory distress syndrome patients. Ann Transl Med 2022; 10:407
84. Chiumello D, Pozzi T, Fratti I, et al. Acid-Base Disorders in COVID-19 Patients with Acute Respiratory Distress Syndrome. J Clin Med 2022; 11
85. Duan J, Chen L, Liu X, et al. An updated HACOR score for predicting the failure of noninvasive ventilation: a multicenter prospective observational study. Crit Care 2022; 26:196
86. Jurjević M, Mirković I, Kopić J, et al. High-PEEP Noninvasive Ventilation By Means of Mask as the Respiratory Support in COVID-19 ARDS Patients: Experience From General Hospital Slavonski Brod. Disaster Med Public Health Prep 2022:1-4
87. Chacko B, Thomas L, Sharma R, et al. Noninvasive Ventilation in the Management of Respiratory Failure Due to COVID-19 Infection: Experience From a Resource-Limited Setting. Mayo Clin Proc 2022; 97:31-45
88. Isaac BTJ, Priya N, Nair AA, et al. Treatment of COVID-19 Acute Respiratory Distress Syndrome With a Tabletop Noninvasive Ventilation Device in a Respiratory Intermediate Care Unit. Mayo Clin Proc Innov Qual Outcomes 2022; 6:239-249
89. Tetaj N, Piselli P, Zito S, et al. Timing and Outcomes of Noninvasive Ventilation in 307 ARDS COVID-19 Patients: An Observational Study in an Italian Third Level COVID-19 Hospital. Medicina (Kaunas) 2022; 58
90. Yaroshetskiy AI, Merzhoeva ZM, Tsareva NA, et al. Breathing pattern, accessory respiratory muscles work, and gas exchange evaluation for prediction of NIV failure in moderate-to-severe COVID-19-associated ARDS after deterioration of respiratory failure outside ICU: the COVID-NIV observational study. BMC Anesthesiol 2022; 22:307


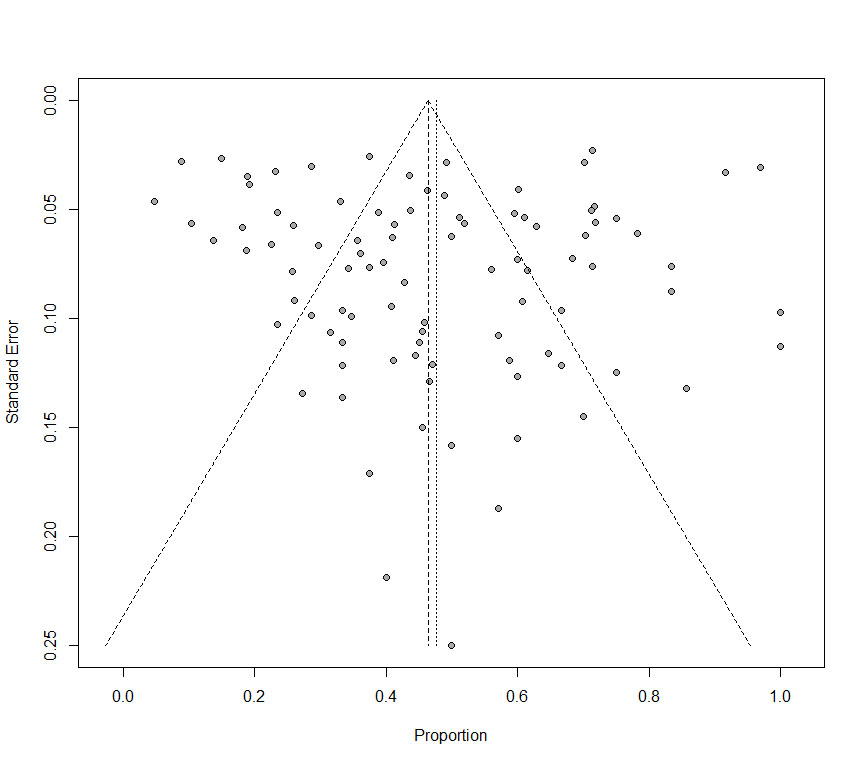


Supplementary Figure 1. Funnel plot of the proportion of NIV failure.


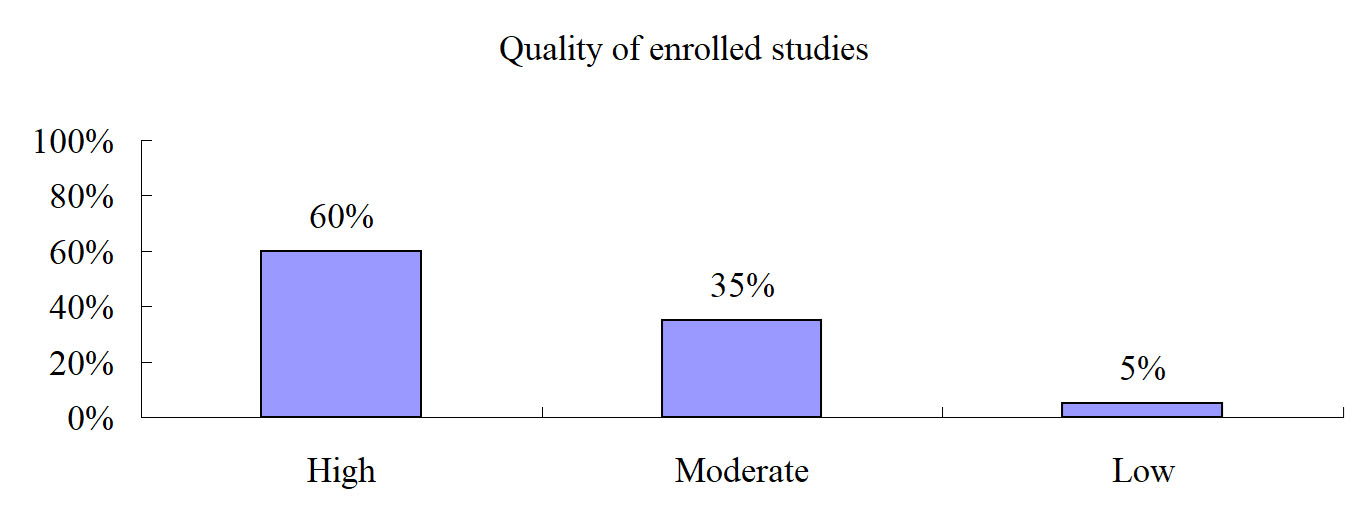


Supplementary Figure 2. Quality assessment of enrolled studies.


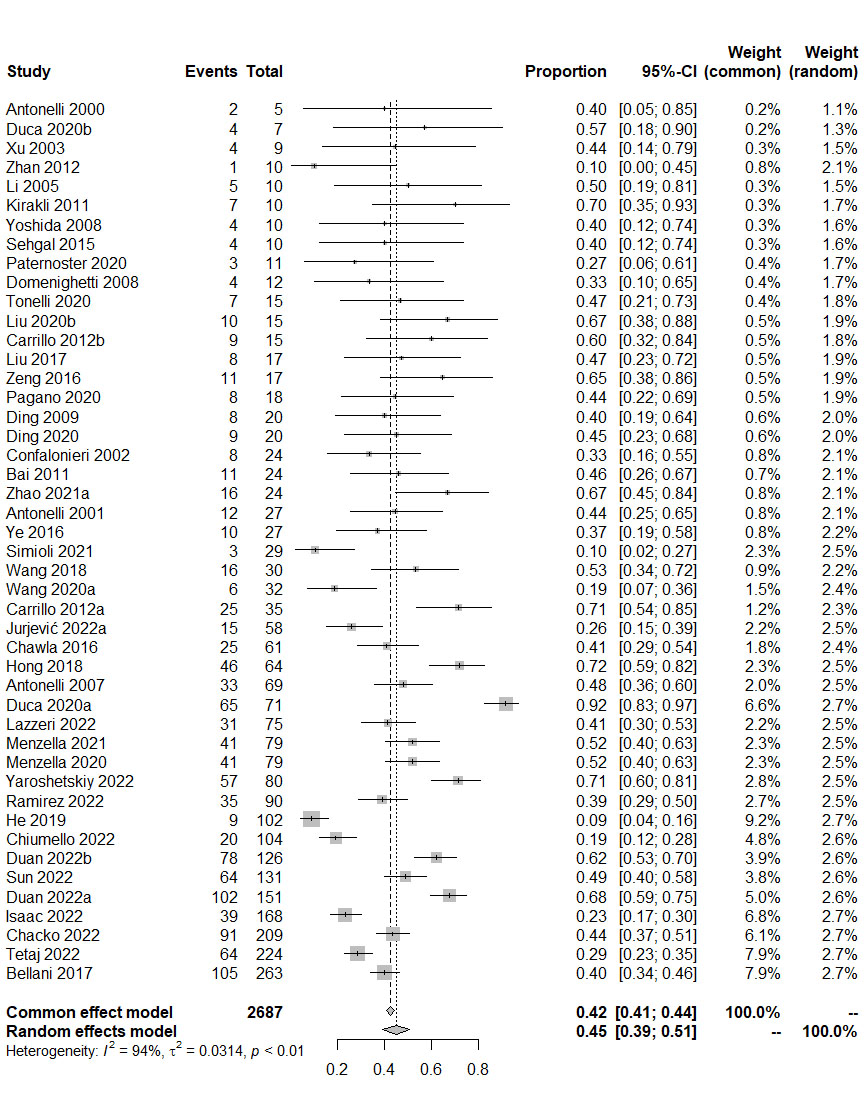


Supplementary Figure 3. Pooled incidence of NIV failure in patients with pulmonary ARDS.


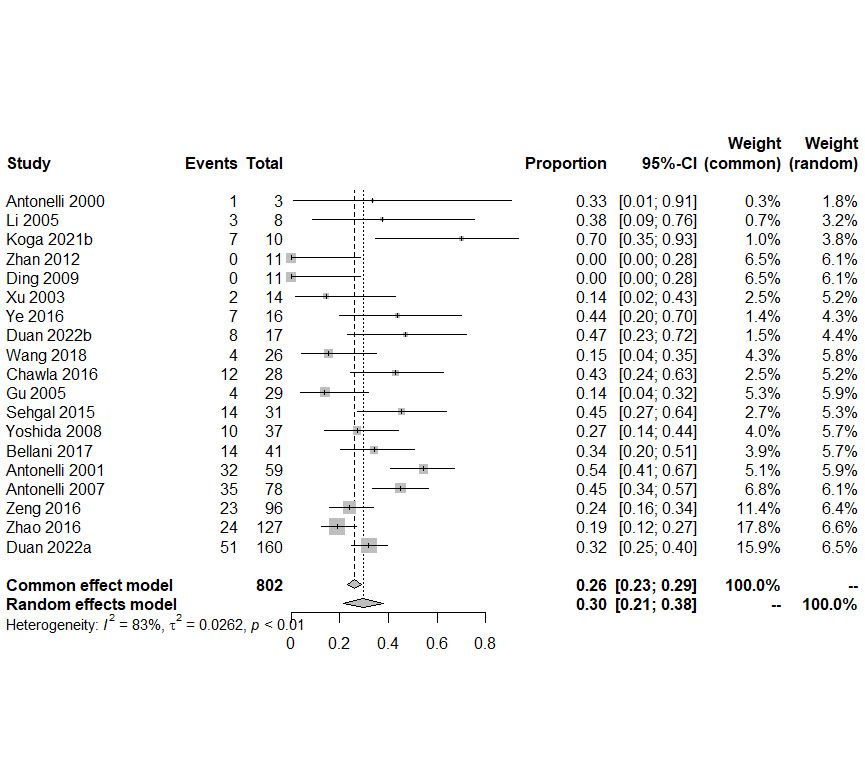


Supplementary Figure 4. Pooled incidence of NIV failure in patients with extra-pulmonary ARDS.


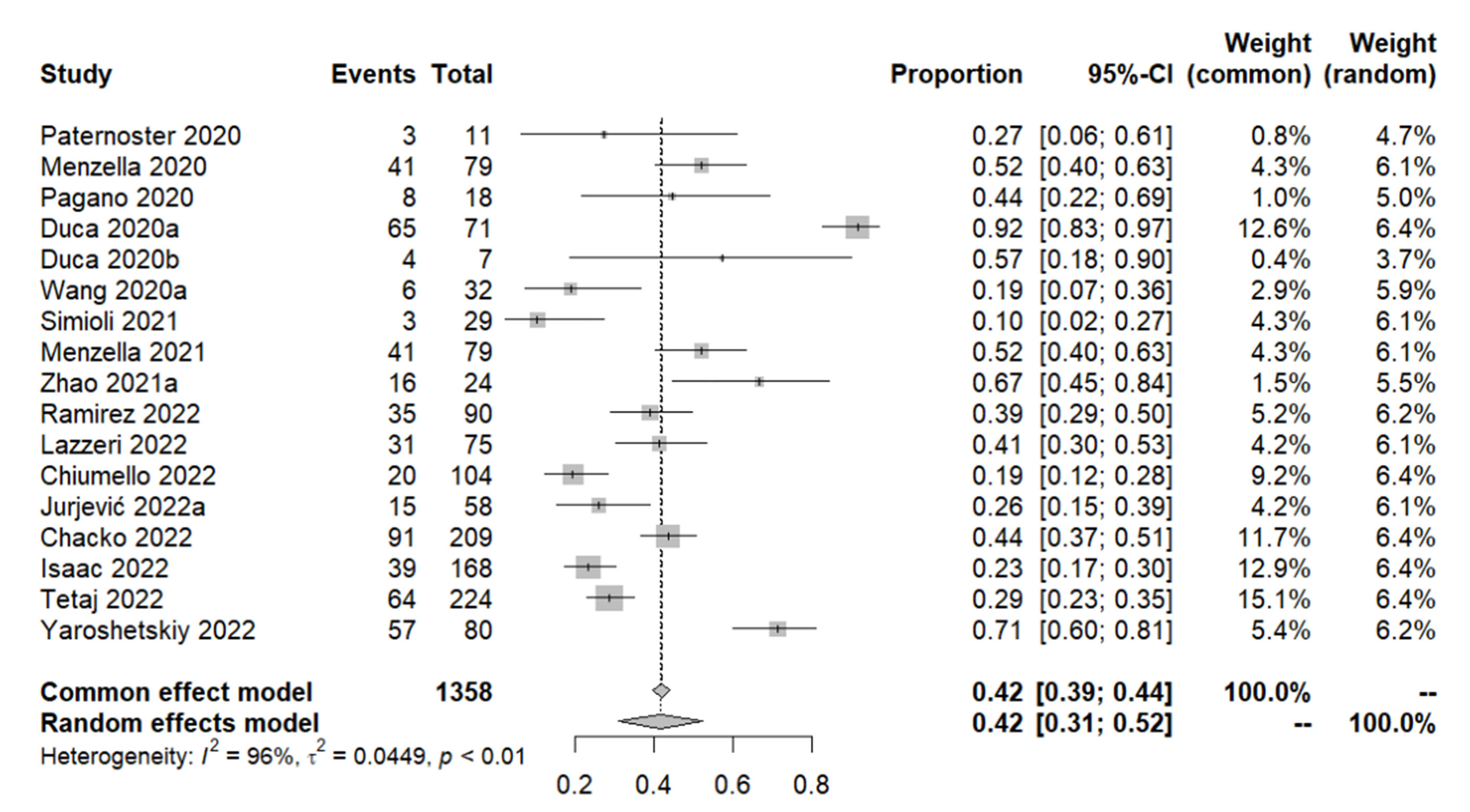


Supplementary Figure 5. Pooled incidence of NIV failure in pulmonary ARDS patients with COVID-19.


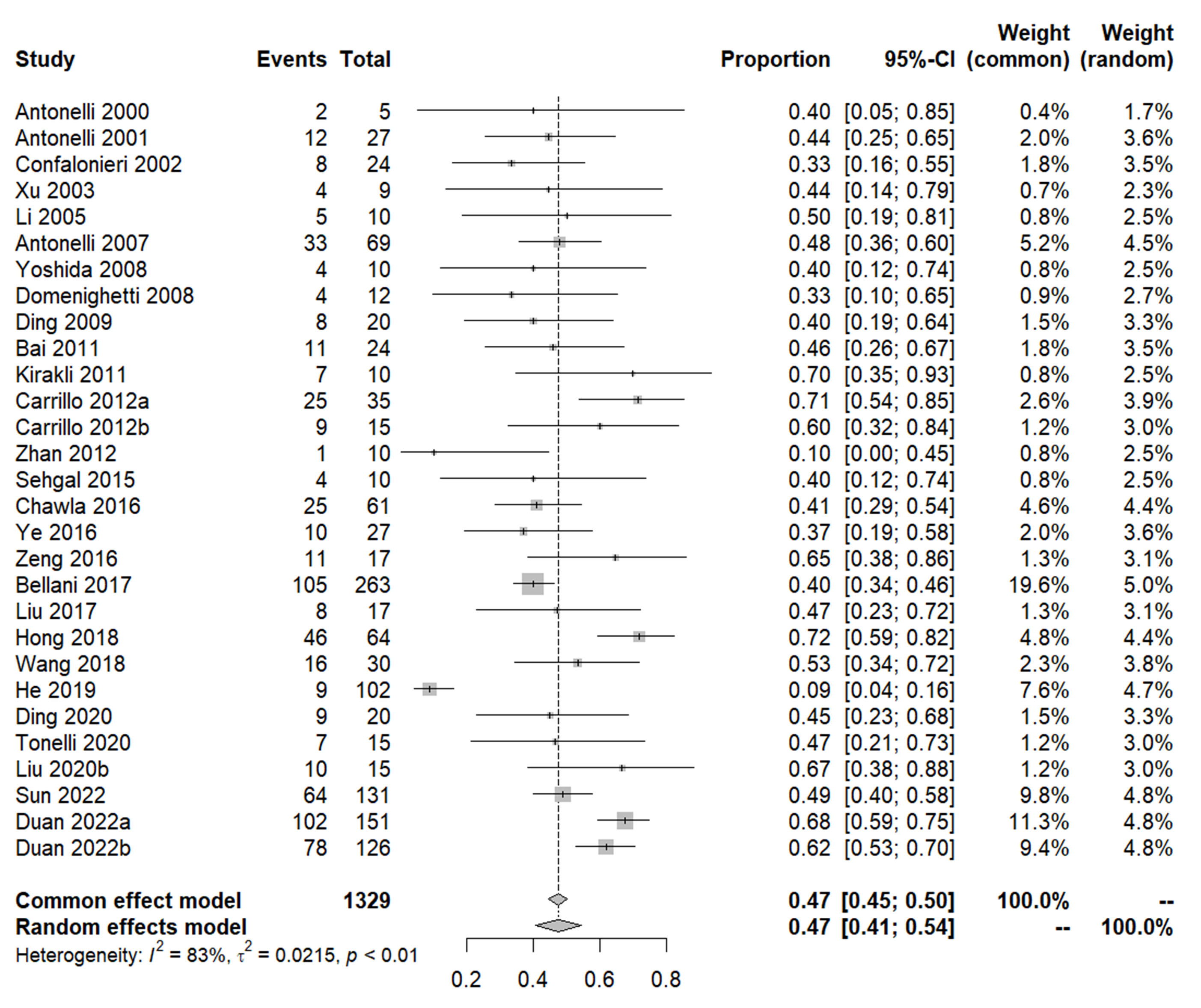


Supplementary Figure 6. Pooled incidence of NIV failure in pulmonary ARDS patients without COVID-19.


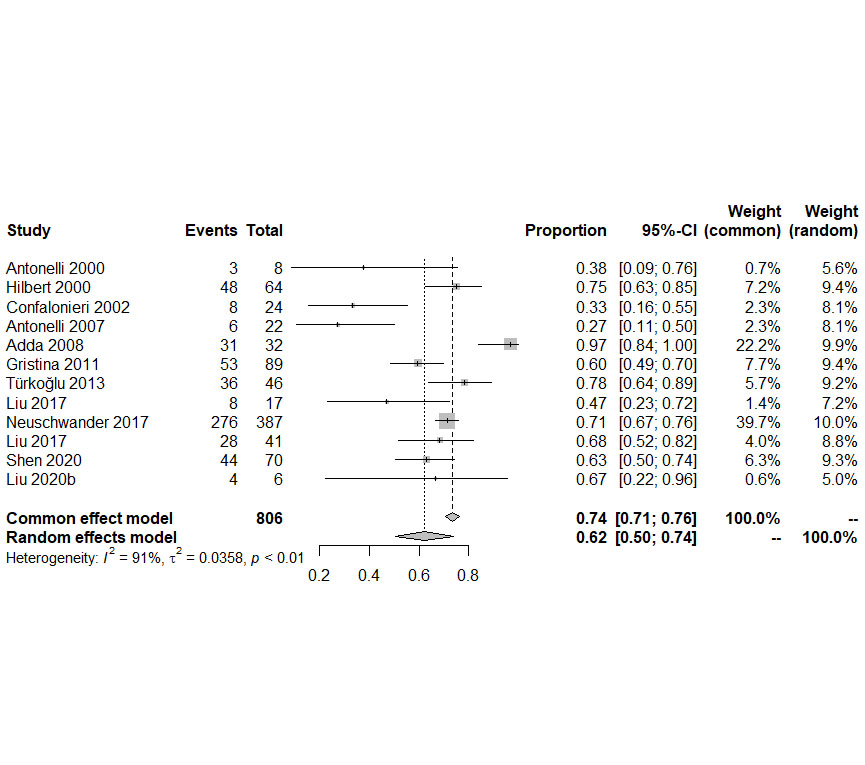


Supplementary Figure 7. Pooled incidence of NIV failure in patients with immunosuppression.


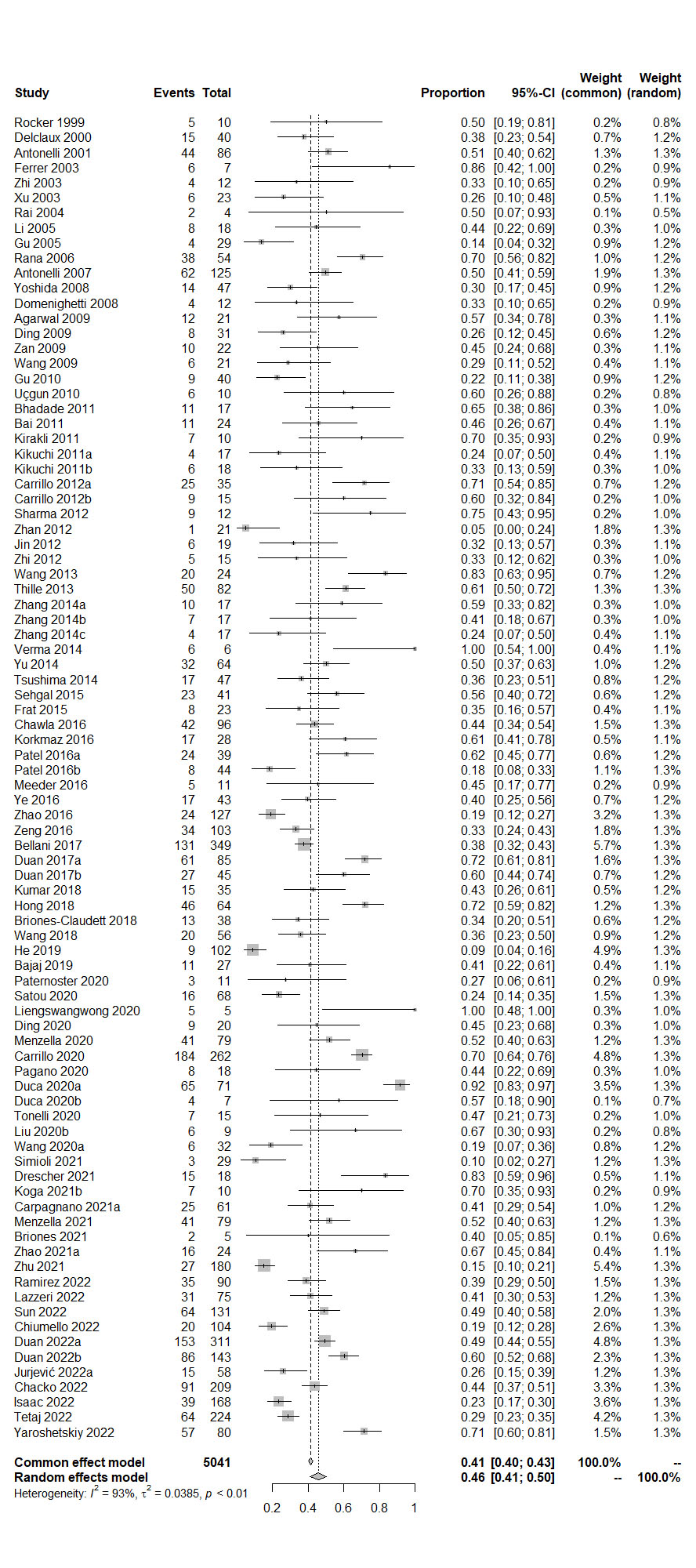


Supplementary Figure 8. Pooled incidence of NIV failure in patients without immunosuppression.


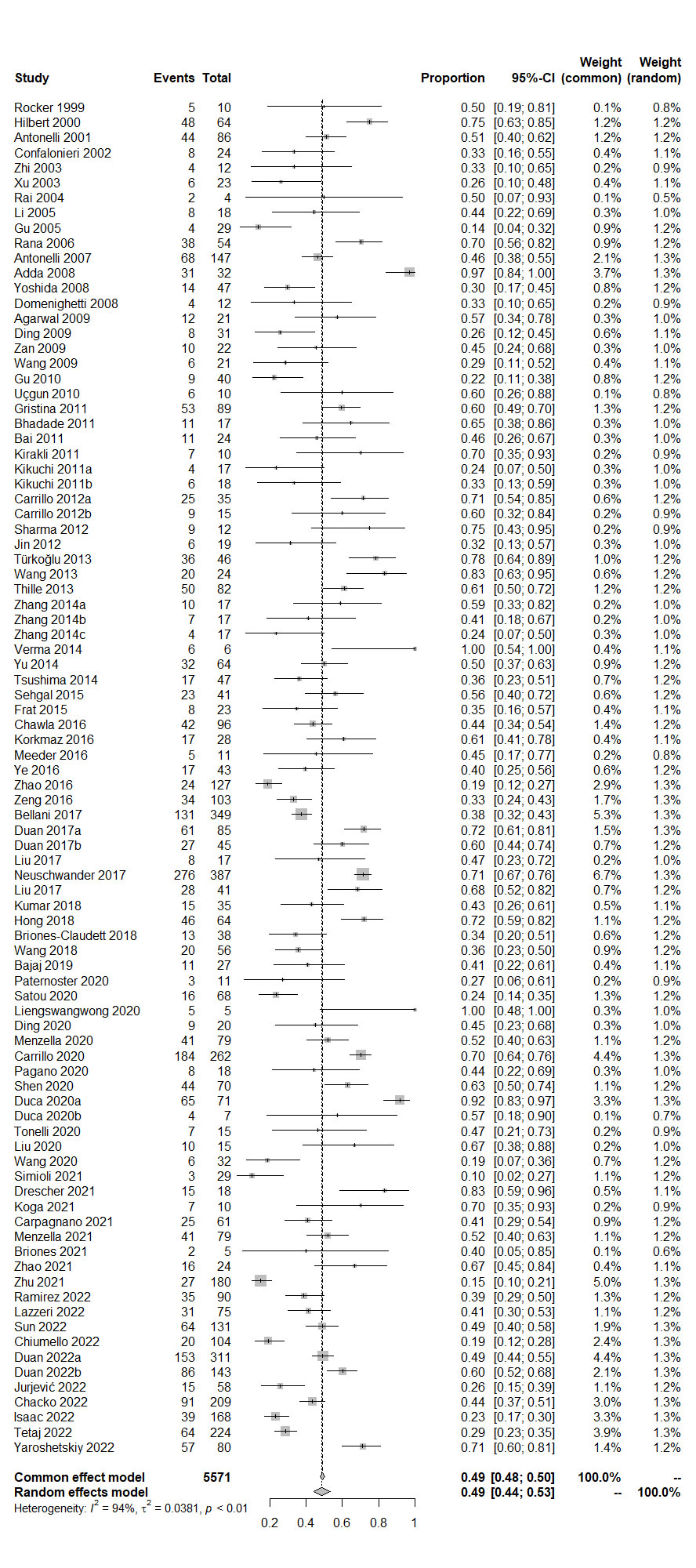


Supplementary Figure 9. Pooled incidence of NIV failure in observational studies.


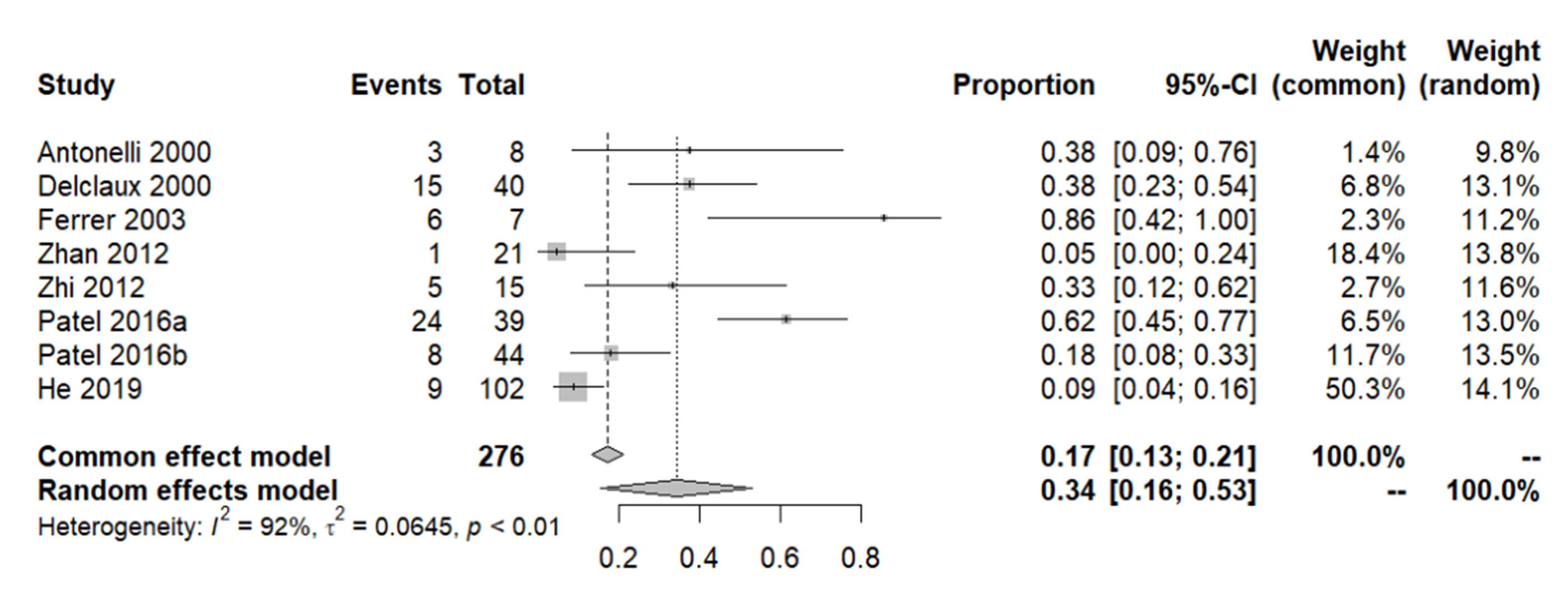


Supplementary Figure 10. Pooled incidence of NIV failure in randomized control trials.


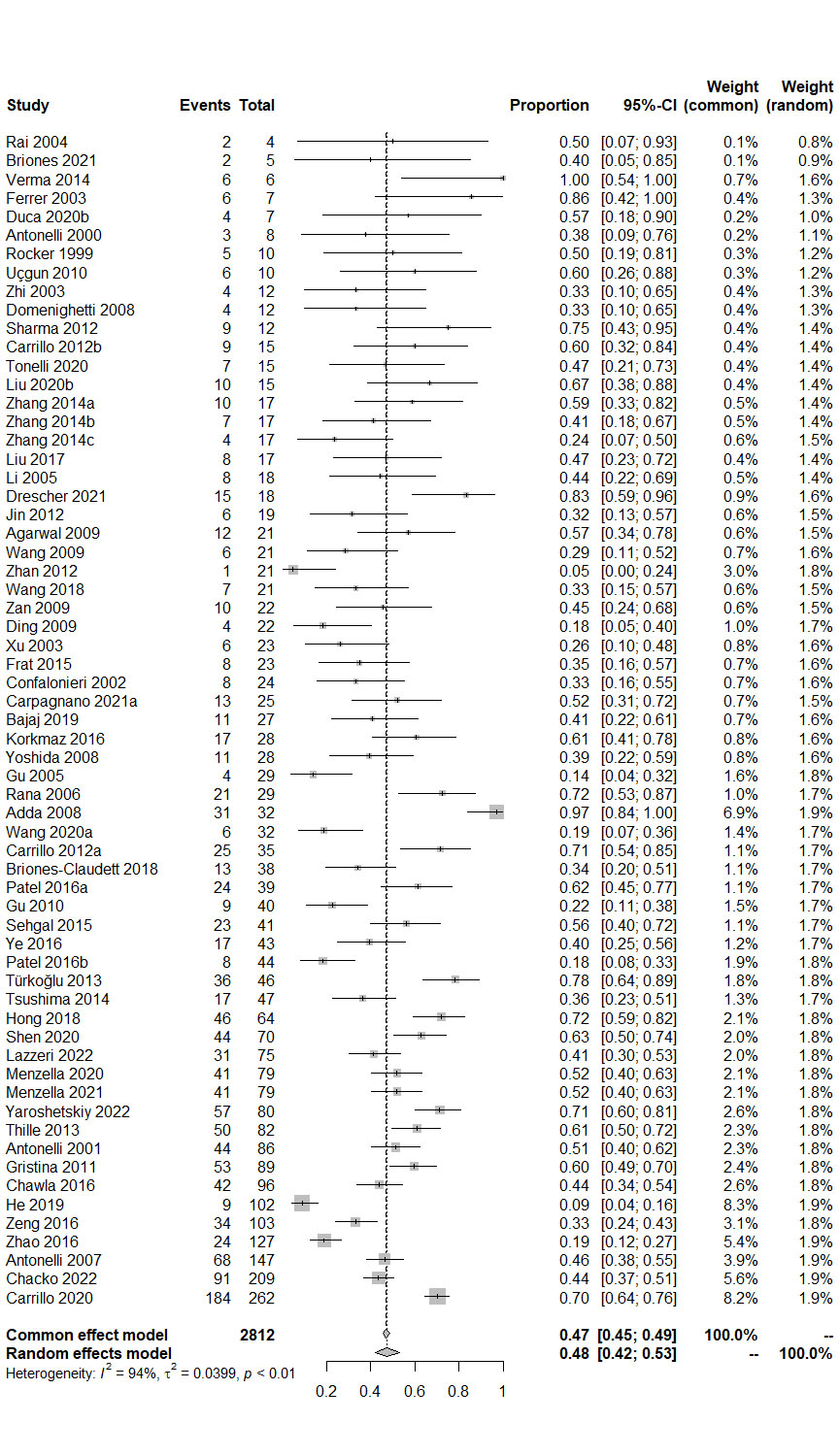


Supplementary Figure 11. Pooled incidence of NIV failure in patients who used BiPAP.


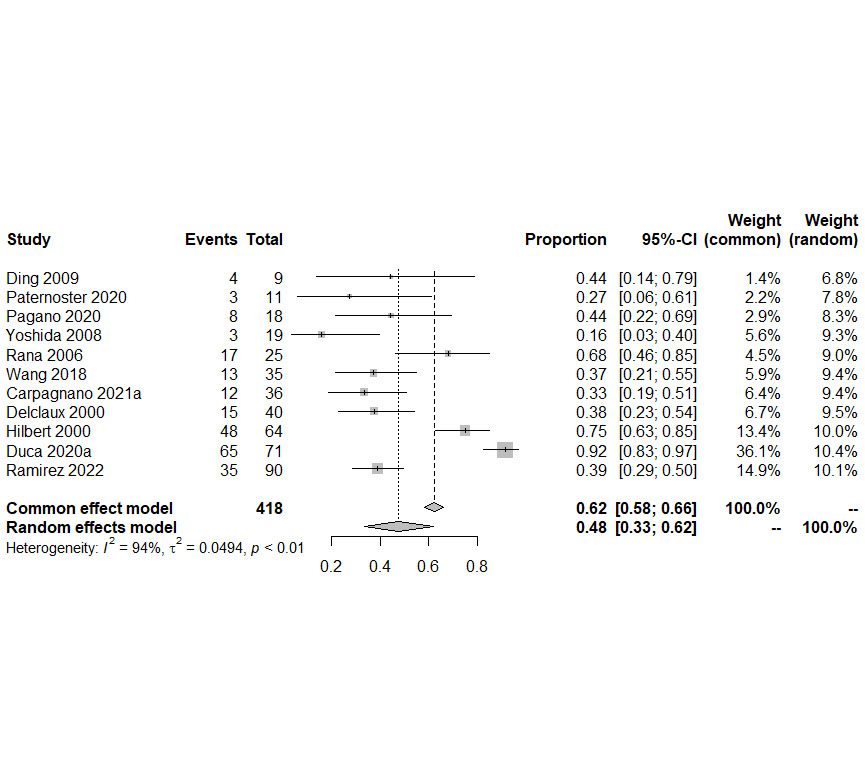


Supplementary Figure 12. Pooled incidence of NIV failure in patients who used CPAP.


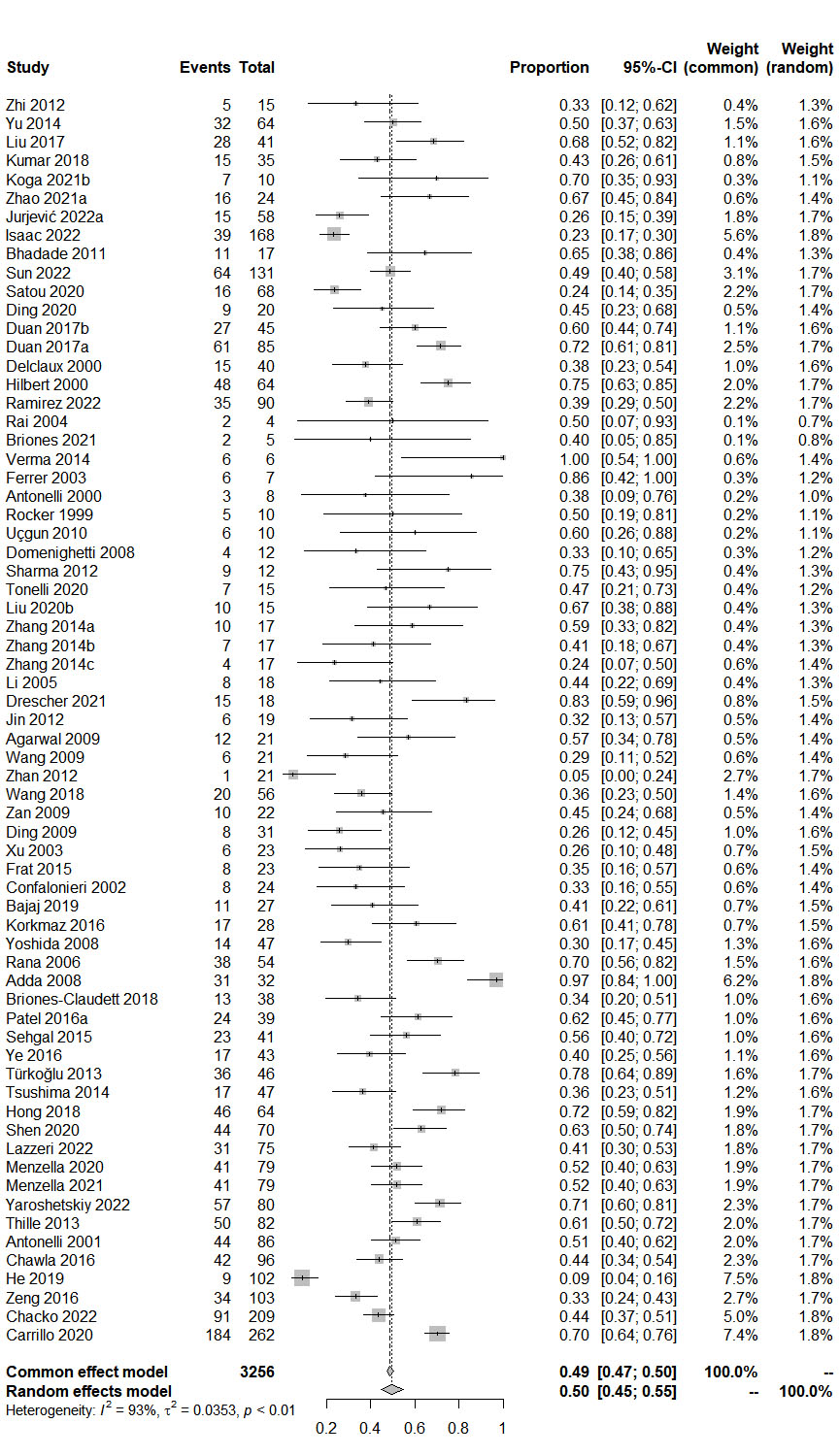


Supplementary Figure 13. Pooled incidence of NIV failure in patients who used an oronasal mask.


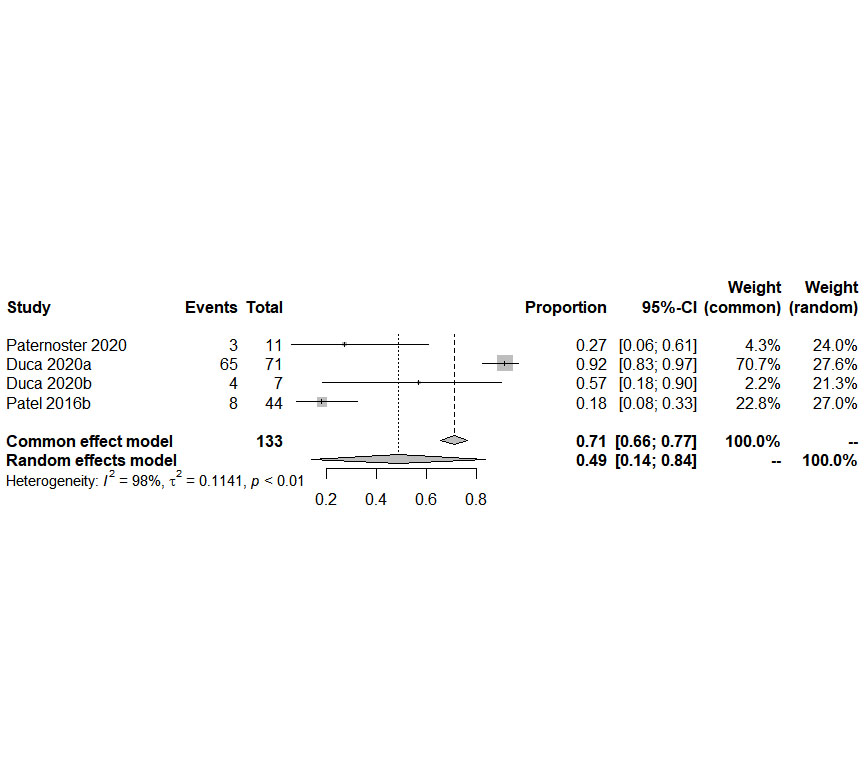


Supplementary Figure 14. Pooled incidence of NIV failure in patients who used a helmet.
